# Pelvic floor muscle training in men with post-prostatectomy urinary incontinence: a scoping review *

**DOI:** 10.1590/1518-8345.7335.4386

**Published:** 2024-10-25

**Authors:** Jackelline Evellin Moreira dos Santos, Virginia Visconde Brasil, Cissa Azevedo, Lívia Cristina de Resende Izidoro, Anna Julia Guimarães Batista, André Carlos Santos Ferreira, Luciana Regina Ferreira da Mata

**Affiliations:** 1 Universidade Federal de Goiás, Faculdade de Enfermagem, Goiânia, GO, Brazil.; ^2^ Universidade Federal de São João del Rei, Curso de Enfermagem, Divinópolis, MG, Brazil.; ^3^ Universidade Federal de Jataí, Curso de Enfermagem, Jataí, GO, Brazil.; ^4^ Universidade Federal de Minas Gerais, Escola de Enfermagem, Belo Horizonte, MG, Brazil.

**Keywords:** Prostatectomy, Urinary Incontinence, Cognitive Behavioral Therapy, Pelvic Diaphragm, Conservative Treatment, Exercise Therapy

## Abstract

**Objective:**

to map pelvic floor muscle training protocols available in the literature for the management of post-prostatectomy urinary incontinence.

**Method:**

this is a scoping review conducted in six databases. Information retrieval was performed using a specific instrument, including: title, authorship, year of publication, journal, objective, study design and description of the protocols’ content.

**Results:**

a total of 24 studies were included, which resulted in different protocols. The most frequent recommendations were the following: three sessions per day; with a frequency of six to 15 contractions per session; performing the exercises in the lying, sitting, and standing positions. The most cited guidelines in the protocols were anal sphincter and bulbocavernosus muscle contractions. All protocols followed the principle of regular pelvic floor muscle contraction, but there was no consensus regarding the start of the protocol, treatment duration, contraction/relaxation time, and intensity of the contraction force.

**Conclusion:**

different orientations were found in the pelvic floor muscle training protocols post-prostatectomy urinary incontinence, with emphasis on contraction time, relaxation time, number of contractions per session, training position, and protocol duration time. Different concepts were adopted to define urinary continence in the protocols. It is necessary to develop consensus guidelines that clearly define the parameters of training protocols.

## Highlights

**(1)** The pelvic floor muscle training is an effective first choice intervention.

**(2)** Scarcity of studies describing the protocols of pelvic floor muscle training detail.

**(3)** There is no consensus between the instructions contained in the different protocols.

## Introduction

 Urinary incontinence is defined as the complaint of any involuntary urine loss ^(^ 2022 ^)^ , being a common complication in men who underwent radical prostatectomy, regardless of the type of surgical procedure ^(^ 2020 ^)^ . 

 Although not yet fully understood, the etiology of post-prostatectomy urinary incontinence is related to decreased or lost integrity, strength and/or activation of the pelvic floor musculature ^(^ 2021 ^-^ 2021 ^)^ . Among the main factors affecting postoperative continence, it can be mentioned those related to the patient (age, obesity and body mass index), biological (preoperative urinary dysfunction, prostate shape and size, and urethral compliance) and surgical factors (bladder neck preservation or reconstruction of urethral support structures) ^(^ 2021 ^-^ 2023 ^)^ . Thus, specific clinical evaluations are necessary to direct the most effective type of treatment according to its etiologic origin ^(^ 2021 ^)^ . 

 Post-prostatectomy urinary incontinence has a wide variation in prevalence (2% to 60%) depending on the methodology used for its assessment ^(^ 2020 ^)^ . It can be a transient condition, with recovery within six months following radical prostatectomy. In some cases symptoms persist 12 months after surgery ^(^ 2019 ^)^ , which has a significant impact on the psychological well-being and quality of life of these individuals ^(^ 2020 ^,^ 2022 ^)^ . 

 In relation to therapeutic management, it is advised to perform the initial management as early as possible ^(^ 2019 ^)^ , conservatively. This modality includes, among other interventions, exercises for the pelvic muscles ^(^ 2020 ^)^ , presented in the literature with the term “pelvic floor muscle training” ^(^ 2023 ^)^ , isolated or combined with electrical stimulation and/or biofeedback ^(^ 2018 ^)^ . 

 The pelvic floor muscle training (PFMT) consists of repeated pelvic muscles contractions in order to improve muscle strength, endurance, and coordination ^(^ 2020 ^,^ 2022 ^)^ . Some of the main muscles involved in male urinary continence in PFMT are the external urethral sphincter, levator ani, and bulbocavernosus, which are activated at different times in coordination ^(^ 2020 ^)^ . 

 No standardized protocol for conducting PFMT in this population has been established in the literature. However, the International Continence Society ^(^ 2023 ^)^ recommends PFMT as the conservative first choice treatment, due to the significant benefits for the continence state and reduced impact on the quality of life of men with post-prostatectomy urinary incontinence ^(^ 2020 ^,^ 2018 ^)^ . 

 It is known that decision-making over the best conduct that ensures care practice includes reviewing the available evidence in the literature. However, the variability of the guidelines provided to men for muscle awareness and proprioception, the exercise program, and the assessment form to verify the correct pelvic floor muscles activation, make it difficult to define effective protocols in clinical practice ^(^ 2020 ^,^ 2019 ^)^ . 

 It is worth noting that it is a low-cost, low-risk, minimally invasive intervention with minimal contraindications ^(^ 2017 ^)^ and with effectiveness evidence of post-prostatectomy urinary incontinence treatment ^(^ 2020 ^)^ . In this context, considering the importance of knowledge regarding PFMT protocols to ensure the continence recovery, this study is justified to synthesize the scope of content found in the programs. 

This study aimed to map the pelvic muscle exercise protocols available in the literature for the management of post-prostatectomy urinary incontinence.

## Method

### Design of the study

 This is a scoping review conducted following the Joanna Briggs Institute scoping reviews ^(^ 2020 ^)^ and reported according to the Preferred Reporting Items for Systematic reviews and Meta-Analyses extension for Scoping Reviews (PRISMA-ScR) checklist ^(^ 2018 ^)^ , in the following steps: definition and allocation of objectives and research questions; inclusion criteria preparation; design and planning of the search strategy and study selection; identification and selection of relevant studies; data extraction and mapping; and results summarization. The review protocol was registered in the Open Science Framework under registration number DOI 10.17605/OSF.IO/HC4ZX. 

### Identification of the research question

To guide the search the research question was prepared following the PCC (Population-Concept-Context) strategy, where P - men with urinary incontinence; C - PFMT protocols for urinary incontinence management; C - post-radical prostatectomy surgery: “What are the pelvic floor muscle training protocols described in the literature for the management of post-prostatectomy urinary incontinence?”.

### Selection criteria

Complete and original primary studies available online in national and international journals, in Portuguese, English, and Spanish, regardless of the publication year, that presented guidelines/protocols related to PFMT for men with post-prostatectomy urinary incontinence were included. Studies that aimed to evaluate the effect of biofeedback or electrostimulation combined with PFMT were included, as long as they presented at least one group submitted to isolated PFMT protocol.

It was excluded editorials, response letters, secondary studies, experience reports or expert opinion; late rehabilitation interventions (start of treatment after one year of radical prostatectomy); PFMT protocols combined with electrostimulation, biofeedback, vibration, and magnetic stimulation; PFMT combined with pharmacological and/or surgical treatment; publications with the same study protocol; case reports and series; and gray literature.

### Search strategy

 A three-step method was used to develop the search strategy ^(^ 2020 ^)^ : 1. Search carried out in two databases to retrieve MeSH terms and keywords; 2. Bibliographic search carried out in virtual databases; 3. Bibliographic search of reference lists. 

The first step included a search in the Medical Literature Analysis and Retrieval System Online (MEDLINE), via the National Library of Medicine (PubMed), and the Virtual Health Library (VHL) website databases, to verify the main descriptors or keywords used in studies regarding the guiding question.

 The selected controlled terms were verified in the *Descritores em Ciências da Saúde* (DeCS) and Medical Subject Heading (MeSH): Prostatectomy, Urinary Incontinence, Behavior Therapy, Pelvic Floor, Muscle, Exercise Therapy, Conservative Treatment, Rehabilitation, Training Support, Contraction, Education and Lifestyle, and Healthy lifestyle. 

The second step was carried out on May 5, 2022, in the following databases: MEDLINE, PubMed; Cochrane Library; SCOPUS, Biomedical Answer (EMBASE); in the VHL website and in the Web of Science (WoS).

**Figure 1:**
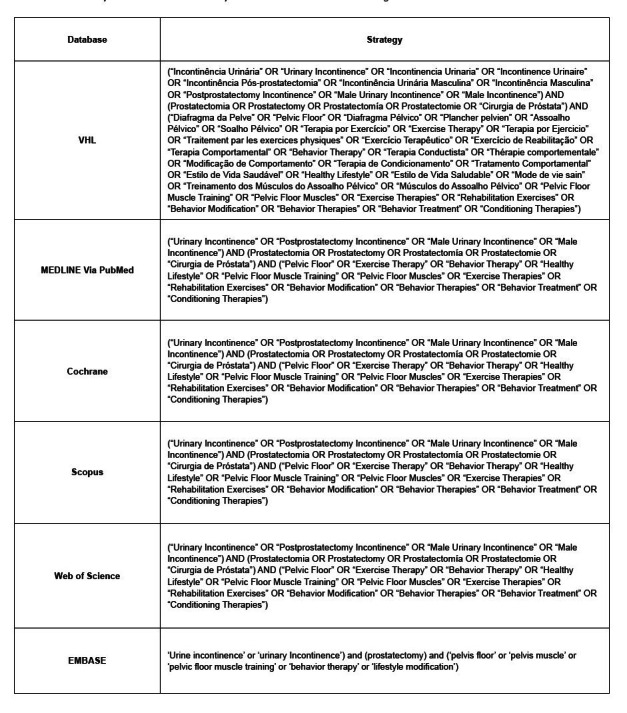
- Search strategies applied and adapted to each database. Goiânia, GO, Brazil, 2022

 It was decided to use controlled descriptors (DeCS and MesH) and keywords (non-controlled descriptors) in Portuguese, English, and Spanish, in order to achieve a targeted search strategy. A single strategy was outlined, which was adapted for each database listed. The Boolean operators AND and OR were used, as shown in Figure 1 . 

### Study selection process

 The EndNote reference manager (version X9 - Desktop) was used to remove duplicates from the results exported from the six databases. The Rayyan platform was used to assist in the process of organizing and selecting primary studies by the reviewers ^(^ 2016 ^)^ . The selection included reading the publications’ titles and abstracts, based on the guiding question and the eligibility criteria. This step was performed by two reviewers independently and blinded. The Rayyan platform blinding was opened and, in consensus meetings, the reviewers selected the studies for full reading, with the participation of a third reviewer. The studies were read in full by two reviewers independently, and in cases of disagreement, a third reviewer was consulted. 

The third step of the search was carried out by manually searching the references of the secondary studies not included to identify studies that met the selection criteria, which had not been previously identified.

### Instruments used to collect information

 To collect and categorize the information, a data extraction tool based on the literature was used ^(^ 2020 ^,^ 2018 ^,^ 2017 ^)^ . The instrument was refined by three nursing researchers with expertise in this field and included the following items: publication title; author(s); publication year; journal; objective; study design; concept adopted for continence and description of the content of the PFMT protocols. 

### Data treatment and analysis

Following categorization, the data were synthesized for descriptive analysis according to their publication year; objective; language; type of study; continence concept and items of the PFMT protocol (time when treatment started, protocol duration, number of sessions per day, number of contractions per session, contraction time, relaxation time, PFMT positions, muscle targeted for contraction, and the way the information was provided).

### Ethical aspects

As it was a scoping review, the research was not submitted for consideration to a Research Ethics Committee.

## Results

**Figure 2:**
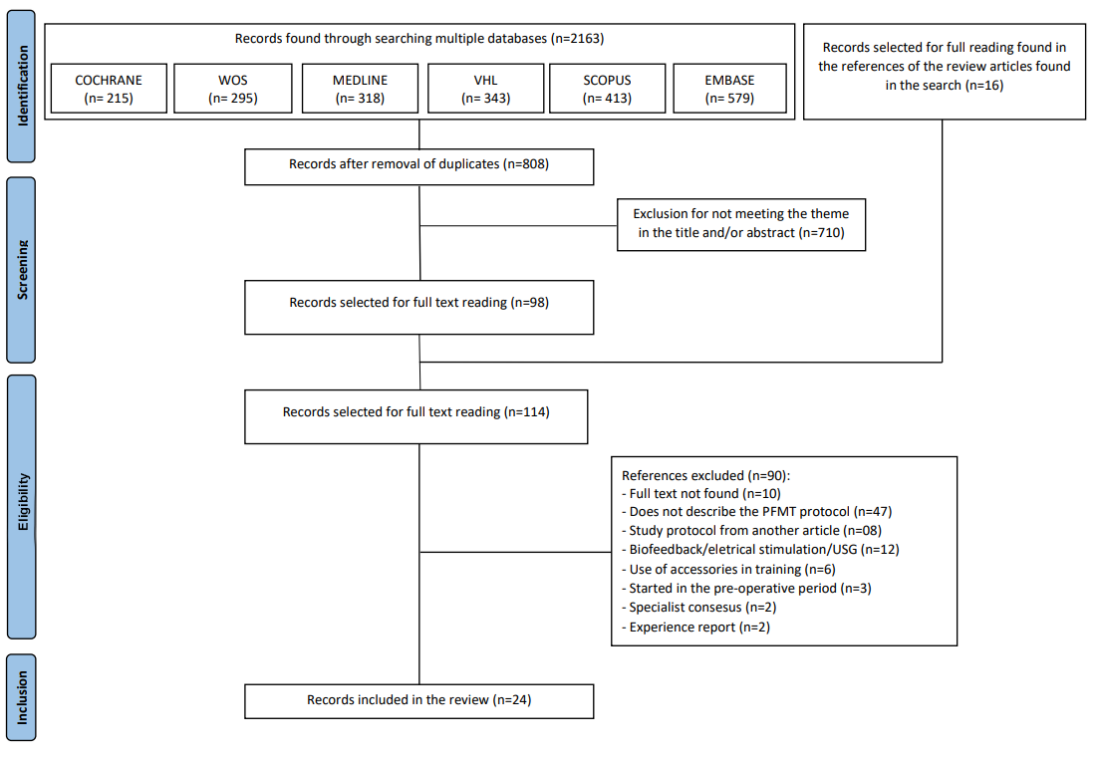
- Flowchart of the selection process of studies included in the scoping review, adapted from the PRISMA Extension for Scoping Reviews (PRISMA-ScR) ^(^ 2018 ^)^ . Goiânia, GO, Brazil, 2022

^*^ PFMT = Pelvic floor muscle training

 A total of 2.163 articles were found in the literature. After title and abstract analysis, 114 articles were read in full and 24 were included in the review ( Figure 2 ). 

**Figure 3:**
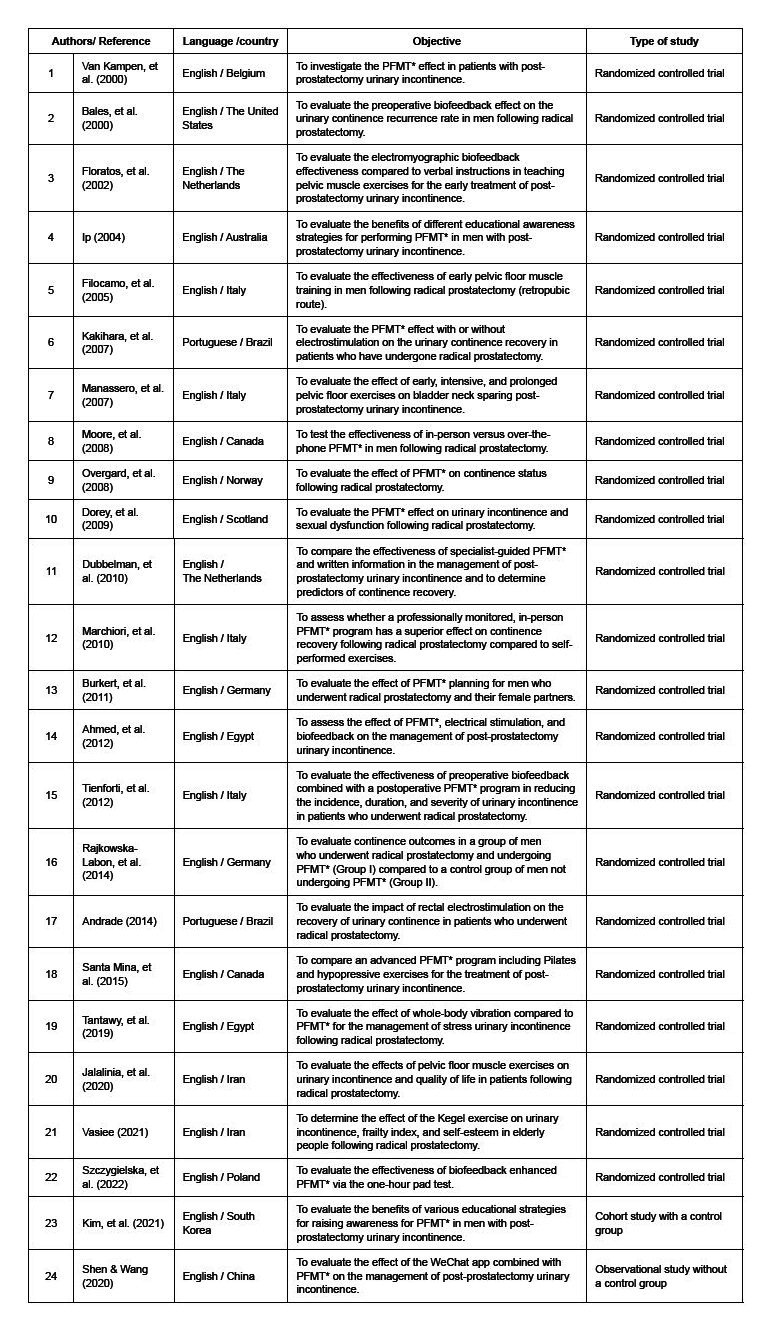
- Classification of the studies included in the mapping of pelvic floor muscle training protocols (n = 24). Goiânia, GO, Brazil, 2022

 The studies were published between 2000 and 2022, with nine (37.5%) being published in the last decade. The prevalent language was English (n=22; 91.7%). The studies were conducted in 15 different countries, with Italy (n=4; 16.7%) having the highest number of publications. In relation to the type of study, 21 (87.5%) were classified as randomized controlled trials, published in 18 different journals ( Figure 3 ). 

 Of the 24 PFMT protocols mapped, four (16.7%) provided a description of the full protocols’ content ^(^ 2004 ^-^ 2008 ^)^ . Three studies (12.5%) addressed or referenced the origin of the instructions presented in the protocol ^(^ 2009 ^,^ 2007 ^-^ 2019 ^)^ . 

 In relation to the urinary continence definition, 10 (41,7%) studies did not provide the conceptualization ^(^ 2004 ^,^ 2009 ^,^ 2007 ^,^ 2014 ^-^ 2010 ^)^ ; three (12,5%) considered continence as the absence of pad use ^(^ 2008 ^-^ 2021 ^)^ ; two (8.33%) considered it as using up to one pad per day ^(^ 2000 ^-^ 2005 ^)^ ; one (4.17%) as urine loss < 10 g ^(^ 2019 ^)^ ; one (4.17%) as urine loss ≤ 8 g within 24 hours ^(^ 2008 ^)^ ; two (8.33%) as urine loss < 1 g by the one-hour pad test ^(^ 2002 ^,^ 2010 ^)^ ; four (16.7%) as urine loss ≤ 2 g in the 24-hour pad test ^(^ 2007 ^-^ 2000 ^)^ ; and one (4.17%) as no urine loss in the 24-hour pad test ^(^ 2020 ^)^ . 

 The most common information reported by the protocols were the following: number of sessions per day (n=22; 91.7%); protocol duration time (n=19; 79.2%); training position (n=19; 79.2%); time when treatment started (n=18; 75%); contraction time (n=18; 75%); and number of contractions per session (n=17; 70.8%). The least described information was the following: the contractions’ intensity (n=22; 91.7%); muscle exercised during contraction (n=12; 50%), and relaxation time (n=11; 45.8%) ( Table 1 ). 

 Most of the information was provided to patients in spoken and written form (n=11; 45.8%), or only in spoken form (n=8; 33.3%) (Table 1). Of the protocols analyzed, eight (33.3%) mentioned the need for instructions on pelvic floor anatomy and/or physiology and on mechanisms related to UI ^(^ 2002 ^-^ 2009 ^,^ 2014 ^,^ 2010 ^-^ 2008 ^,^ 2010 ^,^ 2000 ^-^ 2020 ^)^ . 

In relation to the protocol initiation time, 13 (54.2%) studies recommend starting PFMT up to 15 days following radical prostatectomy. The most frequent PFMT duration in the studies was of up to six months (n=8; 33.3%), with a minimum time of 10 weeks (n=1; 4.17%) and a maximum of 12 months (n=5; 20.8%). Regarding the number of daily sessions, most protocols reported up to three daily sessions (n=18; 75%), in three periods of the day (morning, afternoon, and evening) (Table 1).

The number of contractions per session was one of the most diverse aspects in the literature; seven (29.2%) protocols reported six to 15 contractions, and five protocols (20.8%) reported 15 to 40 contractions. Two (8.33%) studies suggested increasing the number of contractions as the treatment progressed (Table 1).

In relation to the contraction time, seven (29.2%) protocols recommended three to five seconds. As for the relaxation time, four (16.7%) protocols stated six to 10 seconds, with the highest recommended time being 20 seconds (n=2; 8.33%) (Table 1).

In relation to the PFMT performance position, nine (37.5%) protocols mentioned the supine position, sitting, and standing. The orientation to perform PFMT before any effort or activity that may induce UI was mentioned by five (20.8%) protocols (Table 1).

As for the muscles exercised during PFMT, five (20.8%) protocols stated that they focused on the levator ani muscle, and four (12.5%) on the bulbocavernosus muscle (Table 1).

**Table 1:** - Frequency of the items presented in the mapping of pelvic floor muscle training protocols (n = 24). Goiânia, GO, Brazil, 2023

**Protocol items**	**Breakdown of protocol items**	**Frequency** **n (%)**	**Studies found**
**Moment the training starts**	Not mentioned	06 (25.0)	6, 7, 9, 16, 17, 21
	Up to 15 days following radical prostatectomy	13 (54.2)	1, 2, 3, 4, 11, 13, 14, 15, 18, 20, 22, 23, 24
	Up to 30 days following radical prostatectomy	02 (8.33)	7, 8
	> 30 days following radical prostatectomy	03 (12.5)	10, 12, 19
**Duration of the protocol**	Not mentioned	05 (20.8)	1, 9, 11, 16, 19
	Up to 3 months	06 (25.0)	4, 10, 14, 20, 21,22
	Up to 6 months	08 (33.3)	2, 3, 5, 13, 15, 17, 18, 23
	> 6 months	05 (20.8)	6, 7, 8, 12, 24
**Sessions per day**	Not mentioned	02 (8.33)	1, 23
	Up to 3 sessions	18 (75.0)	5, 6, 7, 8, 9, 10, 12, 13, 14, 15, 16, 17, 18, 19, 20, 21, 22, 24
	> than 3 sessions	04 (16.7)	2, 3, 4, 11
**Contractions per session**	Not mentioned	07 (29.2)	1, 5, 13, 15, 16, 20, 23
	06 to 15 contractions	07 (29.2)	2, 4, 9, 10, 11, 12, 24
	12 to 20 contractions	02 (8.33)	8 ,14
	15 to 40 contractions	05 (20.8)	3, 6, 7, 17, 21
	1 ^st^ and 2 ^nd^ week: 15 to 20 / 3 ^rd^ and 4 ^th^ week: 30 to 40 / 5 ^th^ and 6 ^th^ week: 40 to 50 / 7 ^th^ to 26 ^th^ week: 50 to 60	01(4.17)	18
	10 long/ 10 short contractions	01 (4.17)	22
	15 slow/ 20 fast contractions	01 (4.17)	19
**Contraction time**	Not mentioned	06 (25.0)	1, 2, 7, 11, 13, 16
	3 to 5 seconds	07 (29.2)	3, 4, 5, 14, 17, 21, 23
	5 to 10 seconds	02 (8.33)	8, 10
	Starts from 2 to 3 seconds and increases by 1 second daily until it reaches 10 seconds	02 (8.33)	6, 20
	1 ^st^ contraction: 1-2 seconds, alternated with 2 ^nd^ contraction: 6-7 seconds	01 (4.17)	12
	Fast/short/rhythmic fibers: 1 second; Slow/long/sustained fibers: 5 to 10 seconds	06 (25.0)	9, 15, 18, 19, 22, 24
**Relaxation time**	Not mentioned	13 (54.2)	1, 2, 7, 9, 10, 11, 12, 13, 16, 17, 21, 22
	5 seconds	02 (8.33)	4, 20
	Start with 4 seconds of relaxation and increase by 2 seconds daily up to 20 seconds. Then restart the exercises with 4 seconds	01 (4.20)	6
	6 to 10 seconds	04 (16.7)	3, 5, 14, 23
	10 to 20 seconds	01 (4.17)	8
	2 minutes following a set of slow and fast contractions	01 (4.17)	15
	Rhythmic: 1 second / Sustained: 10 seconds	01 (4.17)	18
	Slow fibers: 15 seconds and increase by 1 second every week; fast fibers: 10 seconds	01 (4.17)	19
**Contractions’ intensity**	Not mentioned	22 (91.7)	1, 2, 4, 5, 6, 7, 8, 9, 11, 12, 13, 14, 15, 16, 17, 18, 10, 20, 21, 22, 23, 24
	Maximal (training); submaximal (during activities)	01 (4.17)	10
	70% submaximal strength	01 (4.17)	3
**Training position**	Not mentioned	05 (24.8)	2, 5, 10, 13, 20
	Any position	01 (4.17)	21
	Supine position	03 (12.5)	8, 16, 17
	Supine, sitting, and standing position	09 (37.5)	1, 6, 9, 11, 18, 19, 22, 23, 24
	Supine, sitting, and standing position, as well as before any physical effort or activity that could induce incontinence	05 (20.8)	3, 4, 12, 14, 15
	Initially lateral decubitus, and after getting used it, then sitting and standing positions	01 (4.17)	7
Muscle targeted for contraction*	Not mentioned	11 (45.8)	1, 7, 9, 11, 12, 13, 15, 17, 20, 23, 24
	Anal sphincter / levator ani	05 (20.8)	3, 4, 6, 8, 14
	Striated urethral sphincter	05 (20.8)	2, 16, 19, 21, 22
	Bulbocavernosus muscle	04 (16.7)	5, 8, 10, 18
**How the information was provided to the patient**	Not mentioned	01 (4.17)	5
	Spoken	08 (33.3)	1, 3, 6, 7, 16, 17, 23, 24
	Written	04 (16.7)	4, 11, 13, 21
	Spoken and written	11 (45.8)	2, 8, 9, 10, 12, 14, 15, 19, 20, 22
**Professionals who guided the PFMT** ^†^ **performance**	Not mentioned	04 (16.7)	4, 5, 6, 19
	Physiotherapist	10 (41.7)	1, 3, 10, 11, 13, 14, 16, 17, 18, 22
	Urologist	04 (16.7)	7, 12, 15, 23
	Nurse	04 (16.7)	2, 9, 20, 21
	Nurse or physiotherapist	01 (4.17)	8
	Nurse or urologista	01 (4.17)	24

* A single study may present more than one area as the training target; ^†^ PMFT = Pelvic floor muscle training 

## Discussion

The mapped protocols presented substantial variation in terms of content and diversity of urinary continence definitions.

 The variability in description and the lack of consensus hinder understanding when to start treatment, to establish treatment duration, to define the time of contraction and relaxation, among other important guidelines for continence recovery ^(^ 2022 ^,^ 2017 ^)^ . Corroborating these findings, a systematic review of protocols for the lower urinary tract symptoms management in men confirmed the information variability and the lack of descriptions of the items in the PFMT protocols ^(^ 2018 ^)^ . 

 The lack of reported details can be explained by both by the lack of acknowledgment of the relevance of such information and the word limit imposed by journals ^(^ 2018 ^)^ . The lack of description prevents replicating the study and analysis to assess the effectiveness of the protocol. As an alternative, it would be advisable to use complementary materials to describe the content as appendices or even the publication of the protocol in full, as presented by authors ^(^ 2009 ^)^ . 

 The diversity of definitions presented for the UI phenomenon leads to difficulty in defining the time to recovery of urinary continence. Standardization is needed to ensure that the results of studies can be compared ^(^ 2021 ^,^ 2021 ^)^ . 

 In relation to the professional responsible for conducting PFMT protocols, a prevalence of approaches carried out by physiotherapists was noted. However, in Brazil, other professionals can apply PFMT, such as generalist nurses or specialists in stomatherapy and/or urology, who have the Federal Nursing Council’s support and are considered able to conduct conservative therapies for the post-prostatectomy urinary incontinence treatment ^(^ 2016 ^)^ . 

 The majority of studies have established the need for protocols that lasted longer than three months, however, a shorter duration was considered effective for improving UI in men, in terms of the physiological PFMT effects ^(^ 2019 ^,^ 2022 ^)^ . Effects can be noticed after two weeks, although symptoms decrease more significantly between six and eight weeks of treatment ^(^ 2021 ^)^ . 

 Although a variety of protocols were found, there was consensus on the principle of regularly contracting the pelvic floor muscles in order to increase urethral closure pressure and consequently prevent urine loss. The most cited guidelines were to perform three daily PFMT sessions and to maintain a range of six to 15 contractions per session. Performing 50 to 60 contractions per session between the 7 ^th^ and 26 ^th^ week of follow-up ^(^ 2014 ^)^ , will result in a total of 180 contractions per day. 

 Information on the difficulty progression of the exercises over the course of the weeks was also found in the protocols, starting from the change in positioning, from the easiest level (lying down), to intermediate (sitting), and to the most difficult (standing and while performing daily life activities). It is known that in order to strengthen the pelvic floor, men who underwent radical prostatectomy must become able to exercise the muscles against gravity to support the abdominal contents and prevent urinary loss ^(^ 2021 ^)^ . 

 This is relevant as radical prostatectomy affects not only the urethra and prostate, but also the bladder, moving it lower than where it originally was before surgery ^(^ 2022 ^)^ . As patients become stronger, the muscles may be subjected to greater load and to gravitational forces opposing the pelvic floor elevation ^(^ 2009 ^)^ . 

 The PFMT steps progression may also include increasing the contraction time and decreasing the relaxation time until achieving the number set by the protocol, in order to avoid early fatigue ^(^ 2018 ^,^ 2021 ^)^ . The mean time for pelvic floor muscle fatigue in women with UI is 11.5 seconds ^(^ 2004 ^)^ . Although caution is needed when considering the PFMT evidence in women for men, it is believed that periods of contraction longer than 10 seconds may cause early fatigue in some men ^(^ 2018 ^)^ . 

 On the other hand, it is worth noting that contraction time is associated with gains in muscle strength and endurance ^(^ 2021 ^)^ . Therefore, it is important for patients to increase their contraction time within their capacity to improve urethral resistance ^(^ 2020 ^,^ 2021 ^)^ . Thus, the pelvic floor rehabilitation process entails the individual clinical evaluation of each patient, since when identifying the adequate recognition of the muscles to be exercised and the optimal performance, exercises with a higher difficulty level will be recommended. 

 Another important guideline found in the protocols concerns the performance of a contraction before the increase in intra-abdominal pressure. It is essential for patients to familiarize themselves with the adequate way to perform the exercises, as other irrelevant muscles, such as gluteal and abdominal muscles, may be recruited ^(^ 2022 ^)^ . 

 When recommending PTMT, professionals should explain the anatomy and function of the pelvic floor muscles to patients ^(^ 2020 ^)^ , in order to clarify the recognition of the musculature for the adequate execution of the proposed training. However, most of the studies mapped did not present this information. 

 In this context, there are professionals who mistakenly assume that patients will correctly contract their pelvic floor muscles by receiving only spoken instructions or written materials. However, the isolated use of these strategies without in-person follow-up may lead to poorly performed exercises ^(^ 2021 ^,^ 2023 ^)^ . 

 Another point to be considered is that most studies do not state or reference the source of the instructions used in their interventions. Several protocols apply principles devised for women with stress UI on men, which may compromise the intervention’s effectiveness ^(^ 2020 ^,^ 2018 ^,^ 2021 ^)^ , considering the anatomical differences and variations in the UI mechanism ^(^ 2020 ^)^ . 

 Unlike men, in women the common UI mechanism is the dysfunction of the levator ani muscles, which is secondary to pregnancy and vaginal delivery ^(^ 2011 ^-^ 2008 ^)^ . Thus, PFMT in men should consider the post-prostatectomy urinary incontinence pathophysiology, especially regarding the increased activation of the striated urethral sphincter to compensate for the loss/reduction of the internal sphincter, and maintaining bladder complacence to reduce detrusor overactivity ^(^ 2020 ^,^ 2021 ^)^ . 

 Despite the evidence that the PFMT target in men should be the striated urethral sphincter ^(^ 2020 ^,^ 2018 ^,^ 2021 ^)^ , some studies have targeted the anal sphincter and the bulbocavernosus muscle. These are instructions that may compromise the treatment’s effect, as isolated contraction around the anus does not activate muscles that contract the urethra ^(^ 2020 ^,^ 2021 ^)^ . 

 The information for correct muscle identification is based on attempting to control urination without contracting abdominal muscles, gluteal muscles, and inner thigh muscles. However, it is not clear in the literature the abdominal muscles engagement with pelvic muscles strengthening, as there are hypotheses that the low PFMT effectiveness, in some cases, is associated with the underutilization of abdominal muscles, which may limit the response of the pubococcygeus muscle. This fact justifies the effect of other conservative treatments such as yoga and pilates ^(^ 2020 ^)^ , but the protocols found in this review do not recommend the use of these muscles. 

Due to the specificity and details of the measures described in the PFMT protocols, the relevance of in-person professional monitoring and clarification with easy-to-understand written information, whether in print or digital form, is evident. In addition, for the effective success of the therapeutic plan, it must always be combined with other behavioral interventions that are essential for the gradual continence control, such as eating habits, physical activity, and toilet use.

Even though 80% of the PFMT protocols included in the sample were retrieved from randomized controlled trials, the methodological heterogeneity of the studies limited the possibility of comparing the results.

However, this review’s findings present important clinical and research implications that support the critical sense of healthcare professionals in decision making.

Aspects highlighted in the protocols, such as the contractions’ intensity, the number of sessions, relaxation time and sustained contractions, can be extremely useful in the management of specific cases in clinical practice. In addition, the synthesis of information from the protocols included in this study can significantly contribute to the clinical teaching of these practices in undergraduate and postgraduate health courses. This supports professionals’ critical sense, allowing for more assertive decisions and encouraging evidence-based practice. Integrating these findings into nurses’ training strengthens the application of the best available scientific evidence in clinical decision-making, ensuring more effective and safe interventions.

## Conclusion

This review highlighted that PFMT is an effective, affordable, and non-invasive conventional first choice intervention for the post-prostatectomy urinary incontinence management, which should be started immediately following the removal of the indwelling urinary catheter.

It also provided an overview of the aspects included in PFMT protocols in men with post-prostatectomy urinary incontinence but pointed out the scarcity of studies describing the protocols in detail. Thus, the consensus on which instructions should be followed regarding the start of PFMT, positioning, contraction/relaxation time, contraction intensity, and duration of the protocols is questionable.

In light of this lack of details, careful clinical observation and adequate reporting of the method and results of any future proposal is recommended in the search for the best evidence.

To advance discussions and practices related to post-prostatectomy urinary incontinence, it is necessary to develop consensus guidelines that clearly define the parameters of training protocols. The results obtained from the mapping carried out can support the creation of a list that brings together the practices most cited in the literature, whose effectiveness can be evaluated in future clinical trials, with the aim of standardizing a training protocol for the muscles of the pelvic floor.

## References

[B1] Fernandes A., Sacomani C. A. R., Averbeck M., Prezotti J. A., Ferreira R. S., Moser D. (2022). Tradução para o português Aa international continence society (ICS) report o the terminology for adult neurogenic lower urinary tract dysfunction (ANLUTD). Einstein (São Paulo).

[B2] Hodges P. W., Stafford R. E., Hall L., Neumann P., Morrison S., Frawley H. (2020). Reconsideration of pelvic floor muscle training to prevent and treat incontinence after radical prostatectomy. Urol Oncol.

[B3] Rahnama’i M. S., Marcelissen T., Geavlete B., Tutolo M., Hüsch T. (2021). Current management of post-radical prostatectomy urinary incontinence. Front Surg.

[B4] Mungovan S. F., Carlsson S. V., Gass G. C., Graham P. L., Sandhu J. S., Akin O. (2021). Preoperative exercise interventions to optimize continence outcomes following radical prostatectomy. Nat Rev Urol.

[B5] Gacci M., Nunzio C., Sakalis V., Rieken M., Cornu J. N., Gravas S. (2023). Latest evidence on post-prostatectomy urinary incontinence. J Clin Med.

[B6] O’Connor E., Riogh A., Karavitakis M., Monagas S., Nambiar A. (2021). Diagnosis and non-surgical management of urinary incontinence - a literature review with recommendations for practice. Int J Gen Med.

[B7] Hall L. M., Neumann P., Hodges P. W. (2020). Do features of randomized controlled trials of pelvic floor muscle training for postprostatectomy urinary incontinence differentiate successful from unsuccessful patient outcomes? A systematic review with a series of meta-analyses. Neurourol Urodyn.

[B8] Sandhu J. S., Breyer B., Comiter C., Eastham J. A., Gomez C., Kirages D. J. (2019). Incontinence after Prostate Treatment: AUA/SUFU Guideline. J Urol.

[B9] Ali M. U., Fong K. N. K., Kannan P., Bello U. M., Kranz G. S. (2022). Effects of nonsurgical, minimally or noninvasive therapies for urinary incontinence due to neurogenic bladder: a systematic review and meta-analysis. Ther Adv Chronic Dis.

[B10] Butcher H. K., Dochterman J. M., Bulechek G. M., Wagner C. M. (2020). Classificação das intervenções de enfermagem - NIC.

[B11] Castellan P., Ferretti S., Litterio G., Marchioni M., Schips L. (2023). Management of urinary incontinence following radical prostatectomy: challenges and solutions. Ther Clin Risk Manag.

[B12] Nambiar A. K., Bosch R., Cruz F., Lemack G. E., Thiruchelvam N., Tubaro A. (2018). EAU Guidelines on assessment and nonsurgical management of urinary incontinence. Eur Urol.

[B13] Cardozo L., Rovner E., Wagg A., Wein A., Abrams P. (2023). International Continence Society.

[B14] Hall L. M., Aljuraifani R., Hodges P. W. (2018). Design of programs to train pelvic floor muscles in men with urinary dysfunction: systematic review. Neurourol Urodyn.

[B15] Milios J. E., Ackland T. R., Green D. J. (2019). Pelvic floor muscle training in radical prostatectomy: a randomized controlled trial of the impacts on pelvic floor muscle function and urinary incontinence. BMC Urol.

[B16] Frawley H. C., Dean S. G., Slade S. C., Hay-Smith E. J. C. (2017). Is pelvic-floor muscle training a physical therapy or a behavioral therapy? A call to name and report the physical, cognitive, and behavioral elements. Phys Ther.

[B17] Elrasol Z. M. A., Mohamed O. E. E., Elshhiekh O. G. M. (2020). Effect of pelvic floor muscle strengthening exercises on urinary incontinence and quality of life among patients after prostatectomy. Egypt J Health Care.

[B18] Peters M., Godfrey C., McInerney P., Munn Z., Tricco A., Khalil H., Aromataris E. Munn (2020). JBI Manual for Evidence Synthesis.

[B19] Tricco A. C., Lillie E., Zarin W., O’Brien K. K., Colquhoun H., Levac D. (2018). PRISMA extension for scoping reviews (PRISMA-ScR): checklist and explanation. Ann Intern Med.

[B20] Ouzzani M., Hammady H., Fedorowicz Z., Elmagarmid A. (2016). Rayyan - a web and mobile app for systematic reviews. Syst Rev.

[B21] Abrams P., Cardozo L., Wagg A., Wein  A (2017). Incontinence.

[B22] Ip V (2004). Evaluation of a patient education tool to reduce the incidence of incontinence post-prostate surgery. Urol Nurs [Internet].

[B23] Floratos D. L., Sonke G. S., Rapidou C. A., Alivizatos G. J., Deliveliotis C., Constantinides C. A. (2002). Biofeedback vs verbal feedback as learning tools for pelvic muscle exercises in the early management of urinary incontinence after radical prostatectomy. BJU Int.

[B24] Dorey G., Glazener C., Buckley B., Cochran C., Moore K. (2009). Developing a pelvic floor muscle training regimen for use in a trial intervention. Physiotherapy.

[B25] Moore K. N., Valiquette L., Chetner M. P., Byrniak S., Herbison G. P. (2008). Return to continence after radical retropubic prostatectomy: a randomized trial of verbal and written instructions versus therapist-directed pelvic floor muscle therapy. Urology.

[B26] Kakihara C. T., Sens Y. A. S., Ferreira U. (2007). Effect of functional training for the pelvic floor muscles with or without electrical stimulation in cases of urinary incontinence following radical prostatectomy. Braz J Phys Ther.

[B27] Tantawy S. A., Elgohary H. M. I., Abdelbasset W. K., Kamel D. M. (2019). Effect of 4 weeks of whole-body vibration training in treating stress urinary incontinence after prostate cancer surgery: a randomised controlled trial. Physiotherapy.

[B28] Rajkowska-Labon E., Bakula S., Kucharzewski M., Sliwinski Z. (2014). Efficacy of physiotherapy for urinary incontinence following prostate cancer surgery. Biomed Res Int.

[B29] Szczygielska D., Knapik A., Pop T., Rottermund J., Saulicz E. (2022). The effectiveness of pelvic floor muscle training in men after radical prostatectomy measured with the insert test. Int J Environ Res Public Health.

[B30] Zhang A. Y., Ganocy S., Fu A. Z., Kresevic D., Ponsky L., Strauss G. (2019). Mood outcomes of a behavioral treatment for urinary incontinence in prostate cancer survivors. Support Care Cancer.

[B31] Burkert S., Scholz U., Gralla O., Roigas J., Knoll N. (2011). Dyadic planning of health-behavior change after prostatectomy: a randomized-controlled planning intervention. Soc Sci Med.

[B32] Vasiee A. (2021). Kegel exercise effect on incontinence, Frailty index, and Self-esteem in elderly men after Prostatectomy.

[B33] Jalalinia S. F., Raei M., Naseri-Salahshour V., Varaei S. (2020). The effect of pelvic floor muscle strengthening exercise on urinary incontinence and quality of life in patients after prostatectomy: a randomized clinical trial. Int J Caring Sci.

[B34] Marchiori D., Bertaccini A., Manferrari F., Ferri C., Martorana G. (2010). Pelvic floor rehabilitation for continence recovery after radical prostatectomy: role of a personal training re-educational program. Anticancer Res [Internet].

[B35] Overgard M., Angelsen A., Lydersen S., Morkved S. (2008). Does physiotherapist-guided pelvic floor muscle training reduce urinary incontinence after radical prostatectomy? A randomised controlled trial. Eur Urol.

[B36] Ahmed M. T., Mohammed A. H., Amansour A. (2012). Effect of pelvic floor electrical stimulation and biofeedback on the recovery of urinary continence after radical prostatectomy. Turk J Phys Med Rehab.

[B37] Kim Y. U., Lee D. G., Ko Y. H. (2021). Pelvic floor muscle exercise with biofeedback helps regain urinary continence after robot-assisted radical prostatectomy. J Yeungnam Med Sci.

[B38] Bales G. T., Gerber G. S., Minor T. X., Mhoon D. A., McFarland J. M., Kim H. L. (2000). Effect of preoperative biofeedback/pelvic floor training on continence in men undergoing radical prostatectomy. Urology.

[B39] Filocamo M. T., Li Marzi V., Del Popolo G., Cecconi F., Marzocco M., Tosto A. (2005). Effectiveness of early pelvic floor rehabilitation treatment for post-prostatectomy incontinence. Eur Urol.

[B40] Dubbelman Y., Groen J., Wildhagen M., Rikken B., Bosch R. (2010). The recovery of urinary continence after radical retropubic prostatectomy: a randomized trial comparing the effect of physiotherapist-guided pelvic floor muscle exercises with guidance by an instruction folder only. BJU Int.

[B41] Manassero F., Traversi C., Ales V., Pistolesi D., Panicucci E., Valent F. (2007). Contribution of early intensive prolonged pelvic floor exercises on urinary continence recovery after bladder neck-sparing radical prostatectomy: results of a prospective controlled randomized trial. Neurourol Urodyn.

[B42] Andrade C. E. L. C. (2014). Reabilitação do assoalho pélvico em pacientes com incontinência urinária pós prostatectomia radical [Dissertation].

[B43] D Santa Mina, D Au, SMH Alibhai, L Jamnicky, N Faghani, WJ Hilton (2015). A pilot randomized trial of conventional versus advanced pelvic floor exercises to treat urinary incontinence after radical prostatectomy: a study protocol. BMC Urol.

[B44] M Kampen, W Weerdt, H Poppel, D Ridder, H Feys, L Baert (2000). Effect of pelvic-floor re-education on duration and degree of incontinence after radical prostatectomy: A randomised controlled trial. Lancet.

[B45] Shen J. W., Wang R. J. (2020). The efficacy of the WeChat app combined with pelvic floor muscle exercise for the urinary incontinence after radical prostatectomy. Biomed Res Int.

[B46] Santos N. A., Saintrain M. V., Regadas R. P., Silveira R. A., Menezes F. J. (2017). Assessment of physical therapy strategies for recovery of urinary continence after prostatectomy. Asian Pac J Cancer Prev.

[B47] Nahon I. (2021). Physiotherapy management of incontinence in men. J Physiother.

[B48] Conselho Federal de Enfermagem (BR) (2016). Parecer de Câmara Técnica nº 04/2016/CTAS/COFEN. Manifestação sobre procedimentos da área de enfermagem.

[B49] Izidoro L. C. R., Mata L. R. F., Azevedo C., Paula A. A. P. P., Pereira M. G., Santos J. E. M. (2022). Cognitive-behavioral program to control lower urinary tract symptoms after radical prostatectomy: a randomized clinical trial. Rev Bras Enferm.

[B50] Mohammed A. E., Mohamed M. S. E., Taha S. H., Mohammed R. F. (2021). Educational interventions on reducing stress urinary incontinence episodes among elderly women. Minia Sci Nurs J.

[B51] Cho S. T., Kim K. H. (2021). Pelvic floor muscle exercise and training for coping with urinary incontinence. J Exerc Rehabil.

[B52] Hudolin T., Mitrović H. K., Bakula M., Kuliš T., Penezić L., Zekulić T. (2022). Pelvic rehabilitation for urinary incontinence after radical prostatectomy. Acta Clin Croat.

[B53] Verelst M., Leivseth G. (2004). Are fatigue and disturbances in pre-programmed activity of pelvic floor muscles associated with female stress urinary incontinence?. Neurourol Urodyn.

[B54] Glazener C., Boachie C., Buckley B., Cochran C., Dorey G., Grant A. (2011). Urinary incontinence in men after formal one-to-one pelvic-floor muscle training following radical prostatectomy or transurethral resection of the prostate (MAPS): two parallel randomised controlled trials. Lancet.

[B55] Delancey J. O., Low L. K., Miller J. M., Patel D. A., Tumbarello J. A. (2008). Graphic integration of causal factors of pelvic floor disorders: an integrated life span model. Am J Obstet Gynecol.

[B56] Feng D., Liu S., Li D., Han P., Wei W. (2020). Analysis of conventional versus advanced pelvic floor muscle training in the management of urinary incontinence after radical prostatectomy: a systematic review and meta-analysis of randomized controlled trials. Transl Androl Urol.

