# Supplementary material for: Pelvic floor muscle training in men with post-prostatectomy urinary incontinence: a scoping review
Source: Rev Lat Am Enfermagem. 2024 Oct 25;32:e4386. doi: 10.1590/1518-8345.7335.4386 (PMC11526213; doi:10.1590/1518-8345.7335.4386)
Supplement: Translation

# Translation: Entrenamiento de los músculos del piso pélvico en hombres con incontinencia urinaria post prostatectomía: revisión de alcance*

**DOI:** 10.1590/1518-8345.7335.4385

**Published:** 2024-10-04

**Authors:** Jackelline Evellin Moreira dos Santos, Virginia Visconde Brasil, Cissa Azevedo, Lívia Cristina de Resende Izidoro, Anna Julia Guimarães Batista, André Carlos Santos Ferreira, Luciana Regina Ferreira da Mata

**Affiliations:** 1 Universidade Federal de Goiás, Faculdade de Enfermagem, Goiânia, GO, Brasil.; ^2^ Universidade Federal de São João del Rei, Curso de Enfermagem, Divinópolis, MG, Brasil.; ^3^ Universidade Federal de Jataí, Curso de Enfermagem, Jataí, GO, Brasil.; ^4^ Universidade Federal de Minas Gerais, Escola de Enfermagem, Belo Horizonte, MG, Brasil.

**Keywords:** Prostatectomía, Incontinencia Urinaria, Terapia Cognitivo-Conductual, Diafragma Pélvico, Tratamiento Conservador, Terapia por Ejercicio

## Abstract

mapear los protocolos de ejercicios para los músculos pélvicos disponibles en la literatura para el tratamiento de la incontinencia urinaria post prostatectomía.

esta es una revisión de alcance realizada en seis bases de datos. La recuperación de información se realizó mediante un instrumento específico, que incluye: título, autoría, año de publicación, revista, objetivo, diseño del estudio y descripción del contenido de los protocolos.

se incluyeron 24 estudios en total, de los cuales se obtuvieron diferentes protocolos. Las recomendaciones más frecuentes fueron: tres sesiones por día, con una frecuencia de seis a 15 contracciones por sesión; realizar los ejercicios en posición acostada, sentada y de pie. Las orientaciones más citadas en los protocolos fueron las contracciones del esfínter anal y del músculo bulbocavernoso. Todos los protocolos siguieron el principio de contracción regular de los músculos del piso pélvico, pero no hubo consenso sobre el inicio del protocolo, la duración del tratamiento, el tiempo de contracción/relajación y la intensidad de la fuerza de contracción.

se encontraron diferentes orientaciones en los protocolos de entrenamiento de los músculos del piso pélvico para la incontinencia urinaria post prostatectomía, fundamentalmente sobre el tiempo de contracción, tiempo de relajación, número de contracciones por sesión, posición de entrenamiento y tiempo de duración del protocolo. Se adoptaron diferentes conceptos para definir la continencia urinaria en los protocolos. Es necesario desarrollar guías consensuadas que definan claramente los parámetros de los protocolos de entrenamiento.

## Destacados

**(1)** El entrenamiento de los músculos del piso pélvico es una intervención eficaz de primera elección.

**(2)** Faltan estudios que describan detalladamente los protocolos de entrenamiento de los músculos del piso pélvico.

**(3)** No hay consenso en las instrucciones presentes en los diferentes protocolos.

## Introducción

 La incontinencia urinaria (IU) se define como la queja de cualquier pérdida involuntaria de orina ^(^ 2022 ^)^ , y es una complicación común en hombres sometidos a prostatectomía radical, independientemente del tipo de procedimiento quirúrgico ^(^ 2020 ^)^ . 

 Si bien aún no se comprende completamente, la etiología de la incontinencia urinaria post prostatectomía se relaciona con la disminución o pérdida de la integridad, fuerza y/o activación de los músculos del piso pélvico ^(^ 2021 ^-^ 2021 ^)^ . Entre los principales factores que afectan la continencia postoperatoria se destacan los relacionados con el paciente (edad, obesidad e índice de masa corporal), biológicos (disfunción urinaria preoperatoria, forma y tamaño de la próstata y distensibilidad uretral) y quirúrgicos (preservación del cuello vesical o reconstrucción de las estructuras de soporte uretral) ^(^ 2021 ^-^ 2023 ^)^ . Por lo tanto, es necesario que se realicen evaluaciones clínicas específicas para orientar el tipo de tratamiento más eficaz en función del origen etiológico ^(^ 2021 ^)^ . 

 La prevalencia de la incontinencia urinaria post prostatectomía tiene una amplia variación (2% a 60%) y depende de la metodología utilizada para evaluarla ^(^ 2020 ^)^ . Puede ser una condición transitoria, cuya recuperación se da dentro de los seis meses posteriores a la prostatectomía radical. En algunos casos, los síntomas persisten 12 meses después de la cirugía ^(^ 2019 ^)^ , lo que tiene un impacto significativo en el bienestar psicológico y la calidad de vida del paciente ^(^ 2020 ^,^ 2022 ^)^ . 

 En cuanto al manejo terapéutico, es recomendable realizar un manejo inicial lo más tempranamente posible ^(^ 2019 ^)^ , de forma conservadora. Esta modalidad incluye, entre otras intervenciones, ejercicios para los músculos pélvicos ^(^ 2020 ^)^ , presentes en la literatura con el término “entrenamiento de los músculos del piso pélvico” ^(^ 2023 ^)^ , aislados o combinados con estimulación eléctrica y/o *biofeedback* ^(^ 2018 ^)^ . 

 El entrenamiento de los músculos del piso pélvico (EMPP) consiste en contracciones repetidas de los músculos pélvicos para mejorar la fuerza, resistencia y coordinación muscular ^(^ 2020 ^,^ 2022 ^)^ . Algunos de los principales músculos involucrados en la continencia urinaria masculina en el EMPP son el esfínter uretral externo, el elevador del ano y el bulbocavernoso, que se activan en diferentes momentos de coordinación ^(^ 2020 ^)^ . 

 En la literatura no se ha establecido ningún protocolo estandarizado para realizar el EMPP en esta población. Sin embargo, la *International Continence Society* (Sociedad Internacional de Continencia) ^(^ 2023 ^)^ recomienda el EMPP como tratamiento conservador de primera elección, debido a los importantes beneficios para el estado de continencia y al reducido impacto en la calidad de vida de los hombres con incontinencia urinaria post prostatectomía ^(^ 2020 ^,^ 2018 ^)^ . 

 Se sabe que la toma de decisiones sobre cuál es la mejor conducta para garantizar la práctica del cuidado incluye la revisión de la evidencia disponible en la literatura. Sin embargo, la variabilidad de las directrices para los hombres sobre concientización y propiocepción muscular, el programa de ejercicios y la forma de evaluación para comprobar la correcta activación de los músculos del piso pélvico dificultan la definición de protocolos efectivos en la práctica clínica ^(^ 2020 ^,^ 2019 ^)^ . 

 Cabe resaltar que es una intervención de bajo costo, bajo riesgo, mínimamente invasiva, con contraindicaciones mínimas ^(^ 2017 ^)^ y con evidencia de efectividad para el tratamiento de la incontinencia urinaria post prostatectomía ^(^ 2020 ^)^ . Por consiguiente, considerando la importancia de conocer los protocolos EMPP para garantizar la recuperación de la continencia, se justifica este estudio para resumir el alcance del contenido encontrado en los programas. 

El objetivo de este estudio fue mapear los protocolos de ejercicios para los músculos pélvicos disponibles en la literatura para el tratamiento de la incontinencia urinaria post prostatectomía.

## Método

### Diseño del estudio

 Esta es una revisión de alcance que sigue las directrices para las revisiones de alcance del *Joanna Briggs Institute* ^(^ 2020 ^)^ y fue redactada según la lista de verificación *Preferred Reporting Items for Systematic reviews and Meta-Analyses extension for Scoping Reviews* (PRISMA-ScR) ^(^ 2018 ^)^ . La revisión consta de las siguientes etapas: definición y asignación de objetivos y preguntas de investigación; preparación de criterios de inclusión; concepción y planificación de la estrategia de búsqueda y selección de estudios; identificación y selección de estudios relevantes; extracción y mapeo de datos; y resumen de resultados. El protocolo de revisión fue registrado en el *Open Science Framework* con el número de registro DOI 10.17605/OSF.IO/HC4ZX. 

### Identificación de la pregunta de investigación

Para orientar la búsqueda, se elaboró la pregunta de investigación siguiendo la estrategia PCC (Población-Concepto-Contexto), que corresponde a P - hombres con incontinencia urinaria; C - Protocolos de EMPP para el tratamiento de la incontinencia urinaria; C - cirugía post prostatectomía radical: “¿Cuáles son los protocolos de entrenamiento de los músculos del piso pélvico descritos en la literatura para el tratamiento de la incontinencia urinaria post prostatectomía?”.

### Criterios de selección

 Se incluyeron estudios primarios completos y originales disponibles *online* en revistas nacionales e internacionales, en portugués, inglés y español, independientemente del año de publicación, que presentaran directrices/protocolos relacionados con el EMPP para hombres con incontinencia urinaria post prostatectomía. Se incluyeron los estudios cuyo objetivo era evaluar el efecto de la *biofeedback* o estimulación eléctrica combinada con EMPP, siempre que presentaran al menos un grupo sometido a un protocolo aislado de EMPP. 

 Se excluyeron editoriales, cartas de respuesta, estudios secundarios, relatos de experiencia u opiniones de expertos; intervenciones de rehabilitación tardías (inicio del tratamiento un año después de la prostatectomía radical); protocolos de EMPP combinados con estimulación eléctrica, *biofeedback* , vibración y estimulación magnética; EMPP combinados con tratamiento farmacológico y/o quirúrgico; publicaciones con el mismo protocolo de estudio; informes y series de casos; y literatura gris. 

### Estrategia de investigación

 Se utilizó un método que consta de tres etapas para desarrollar la estrategia de búsqueda ^(^ 2020 ^)^ : 1. Búsqueda realizada en dos bases de datos para recuperar términos MeSH y palabras clave; 2. Búsqueda bibliográfica realizada en bases de datos virtuales; 3. Búsqueda bibliográfica de listas de referencias. 

 La primera etapa incluyó una búsqueda en el *Medical Literature Analysis and Retrieval System Online* (MEDLINE), a través de la *National Library of Medicine* (PubMed), y en las bases de datos del sitio *web* de la Biblioteca Virtual en Salud (BVS), para verificar los principales descriptores o palabras clave utilizados en los estudios relacionados con la pregunta orientadora. 

 Los términos controlados seleccionados se verificaron en los Descriptores de Ciencias de la Salud (DeCS) y en el *Medical Subject Heading* (MeSH): Prostatectomía, incontinencia urinaria, terapia conductual, piso pélvico, músculos, terapia de ejercicio, tratamiento conservador, rehabilitación, apoyo al entrenamiento, contracción, educación y estilo de vida y estilo de vida saludable. 

 La segunda etapa se realizó el 5 de mayo de 2022, en las siguientes bases de datos: MEDLINE, PubMed; Biblioteca *Cochrane* ; SCOPUS, *Biomedical Answer* (EMBASE); en el sitio *web* de la BVS y en *Web of Science* (WoS). 

 Se decidió utilizar descriptores controlados (DeCS y MesH) y palabras clave (descriptores no controlados) en portugués, inglés y español para realizar una estrategia de búsqueda dirigida. Se diseño una sola estrategia, que se adaptó para cada una de las mencionadas bases datos. Se utilizaron los operadores booleanos AND y OR, como se muestra en la Figura 1 . 

**Figura 1:** - Estrategias de búsqueda aplicadas y adaptadas a cada base de datos. Goiânia, GO, Brasil, 2022

### Proceso de selección de estudios

 Se utilizó el administrador de referencias *EndNote* (versión X9 - *Desktop* ) para eliminar duplicados de los resultados exportados de las seis bases de datos. Se utilizó la plataforma *Rayyan* para ayudar a los revisores en el proceso de organización y selección de los estudios primarios ^(^ 2016 ^)^ . La selección incluyó la lectura de los títulos y resúmenes de las publicaciones, y se basó en la pregunta orientadora y los criterios de elegibilidad. Este paso fue realizado por dos revisores de forma independiente y ciega. El cegamiento de la plataforma *Rayyan* fue abierto y, en reuniones de consenso, los revisores seleccionaron los estudios para lectura completa, con la participación de un tercer revisor. Dos revisores leyeron los estudios completos de forma independiente y, en casos de desacuerdo, se consultó a un tercer revisor. 

La tercera etapa de la investigación se realizó mediante la búsqueda manual de las referencias de estudios secundarios no incluidos para identificar estudios que cumplieran con los criterios de selección y que no hubieran sido identificados previamente.

### Instrumentos utilizados para recopilar información

 Para recopilar y categorizar la información se utilizó una herramienta de extracción de datos basada en la literatura ^(^ 2020 ^,^ 2018 ^,^ 2017 ^)^ . El instrumento fue perfeccionado por tres investigadores de enfermería con experiencia en ese campo e incluyó los siguientes ítems: título de la publicación; autor(es); año de publicación; revista; objetivo; diseño del estudio; concepto adoptado para continencia y descripción del contenido de los protocolos del EMPP. 

### Procesamiento y análisis de datos

Luego de la categorización, los datos fueron sintetizados para realizar el análisis descriptivo según año de publicación, objetivo, idioma, tipo de estudio, concepto de continencia e ítems del protocolo de EMPP (hora de inicio del tratamiento, duración del protocolo, número de sesiones por día, número de contracciones por sesión, tiempo de contracción, tiempo de relajación, posiciones del EMPP, músculo objetivo de la contracción y forma en que se proporcionó la información).

### Aspectos éticos

Dado que se trata de un análisis de alcance, la investigación no fue sometida a la evaluación de un Comité de Ética en Investigación.

## Resultados

 Se encontraron en la literatura 2.163 artículos en total. Después de analizar el título y el resumen, se leyeron 114 artículos completos y 24 se incluyeron en la revisión ( Figura 2 ). 

 Los estudios fueron publicados entre 2000 y 2022, nueve de ellos (37,5%) fueron publicados en la última década. El idioma predominante fue el inglés (n=22; 91,7%). Los estudios se llevaron a cabo en 15 países diferentes, Italia (n=4; 16,7%) fue el que tuvo el mayor número de publicaciones. En cuanto al tipo de estudio, 21 (87,5%) fueron clasificados como ensayos clínicos aleatorizados, publicados en 18 revistas diferentes ( Figura 3 ). 

 De los 24 protocolos de EMPP mapeados, cuatro (16,7%) proporcionaban una descripción del contenido completo de los protocolos ^(^ 2004 ^-^ 2008 ^)^ . Tres estudios (12,5%) abordaban o hacían referencia al origen de las instrucciones presentadas en el protocolo ^(^ 2009 ^,^ 2007 ^-^ 2019 ^)^ . 

 En cuanto a la definición de continencia urinaria, 10 (41,7%) estudios no incluían el concepto ^(^ 2004 ^,^ 2009 ^,^ 2007 ^,^ 2014 ^-^ 2010 ^)^ ; tres (12,5%) consideraban la continencia como la ausencia del uso de toallas absorbentes ^(^ 2008 ^-^ 2021 ^)^ ; dos (8,33%) consideraban el uso de hasta una toalla absorbente por día; uno (4,17%) como pérdida de orina < 10 g ^(^ 2019 ^)^ ; uno (4,17%) como pérdida de orina ≤ 8 g en 24 horas ^(^ 2008 ^)^ ; dos (8,33%) como pérdida de orina < 1 g en la prueba de absorbente de una hora ^(^ 2002 ^,^ 2010 ^)^ ; cuatro (16,7%) como pérdida de orina ≤ 2 g en la prueba de absorbente de 24 horas ^(^ 2007 ^-^ 2000 ^)^ ; y uno (4,17%) como ausencia de pérdida de orina en la prueba de absorbente de 24 horas ^(^ 2020 ^)^ . 

**Figura 2:** - Diagrama de flujo del proceso de selección de los estudios incluidos en la revisión de alcance, adaptado de PRISMA *Extension for Scoping Reviews* (PRISMA-ScR) ^(^ 2018 ^)^ . Goiânia, GO, Brasil, 2022

**Figura 3:** - Clasificación de los estudios incluidos en el mapeo de protocolos de entrenamiento de los músculos del piso pélvico (n = 24). Goiânia, GO, Brasil, 2022

^*^ EMPP = Entrenamiento de los músculos del piso pélvico

 La información más común presente en los protocolos fue: número de sesiones por día (n=22; 91,7%); duración del protocolo (n=19; 79,2%); posición de entrenamiento (n=19; 79,2%); hora de inicio del tratamiento (n=18; 75%); tiempo de contracción (n=18; 75%); y número de contracciones por sesión (n=17; 70,8%). Los datos menos descritos fueron: intensidad de las contracciones (n=22; 91,7%); músculo ejercitado durante la contracción (n=12; 50%) y tiempo de relajación (n=11; 45,8%) ( Tabla 1 ). 

 La mayor parte de la información se les proporcionó a los pacientes de forma oral y escrita (n=11; 45,8%), o solo de forma oral (n=8; 33,3%) ( Tabla 1 ). Ocho (33,3%) de los protocolos analizados mencionaron que era necesario brindar información sobre la anatomía y/o fisiología del piso pélvico y sobre los mecanismos relacionados con la IU ^(^ 2002 ^-^ 2009 ^,^ 2014 ^,^ 2010 ^-^ 2008 ^,^ 2010 ^,^ 2000 ^-^ 2020 ^)^ . 

 En cuanto al momento de inicio del protocolo, 13 (54,2%) estudios recomiendan comenzar el EMPP dentro de los 15 días posteriores a la prostatectomía radical. La duración más frecuente del EMPP en los estudios fue de hasta seis meses (n=8; 33,3%), con un tiempo mínimo de 10 semanas (n=1; 4,17%) y un máximo de 12 meses (n= 5; 20,8%). Con respecto al número de sesiones diarias, la mayoría de los protocolos mencionaron hasta tres sesiones diarias (n=18; 75%), en tres períodos del día (mañana, tarde y noche) ( Tabla 1 ). 

 El número de contracciones por sesión fue uno de los aspectos que presentó mayor divergencia en la literatura; siete (29,2%) protocolos informaron de seis a 15 contracciones y cinco protocolos (20,8%) informaron de 15 a 40 contracciones. Dos (8,33%) estudios sugirieron aumentar el número de contracciones a medida que avanzaba el tratamiento ( Tabla 1 ). 

 En lo que respecta al tiempo de contracción, siete (29,2%) protocolos recomendaron de tres a cinco segundos. En cuanto al tiempo de relajación, cuatro (16,7%) protocolos indicaron de seis a 10 segundos, y el tiempo más largo recomendado fue 20 segundos (n=2; 8,33%) ( Tabla 1 ). 

 Con respecto a la posición para la realización del EMPP, nueve (37,5%) protocolos mencionaron las posiciones: supina, sentada y de pie. La recomendación de realizar el EMPP antes de cualquier esfuerzo o actividad que pudiera inducir IU fue mencionada en cinco (20,8%) protocolos ( Tabla 1 ). 

 En cuanto a los músculos ejercitados durante el EMPP, cinco (20,8%) protocolos mencionaron que se enfocaban en el músculo elevador del ano y cuatro (12,5%) en el músculo bulbocavernoso ( Tabla 1 ). 

**Tabla 1:** - Frecuencia de los ítems presentados en el mapeo de protocolos de entrenamiento de los músculos del piso pélvico (n = 24). Goiânia, GO, Brasil, 2023

Ítems **del protocolo**	**Detalles de los ítems del protocolo**	**Frecuencia** **n (%)**	**Estudios realizados**
**Cuando comienza el entrenamiento**	No mencionado	06 (25,0)	6, 7, 9, 16, 17, 21
	Hasta 15 días después de la prostatectomía radical	13 (54,2)	1, 2, 3, 4, 11, 13, 14, 15, 18, 20, 22, 23, 24
	Hasta 30 días después de la prostatectomía radical	02 (8,33)	7, 8
	> 30 días después de la prostatectomía radical	03 (12,5)	10, 12, 19
**Duración del protocolo**	No mencionado	05 (20,8)	1, 9, 11, 16, 19
	Hasta 3 meses	06 (25,0)	4, 10, 14, 20, 21,22
	Hasta 6 meses	08 (33,3)	2, 3, 5, 13, 15, 17, 18, 23
	> 6 meses	05 (20,8)	6, 7, 8, 12, 24
**Sesiones por día**	No mencionado	02 (8,33)	1, 23
	Hasta 3 sesiones	18 (75,0)	5, 6, 7, 8, 9, 10, 12, 13, 14, 15, 16, 17, 18, 19, 20, 21, 22, 24
	> de 3 sesiones	04 (16,7)	2, 3, 4, 11
**Contracciones por sesión**	No mencionado	07 (29,2)	1, 5, 13, 15, 16, 20, 23
	06 a 15 contracciones	07 (29,2)	2, 4, 9, 10, 11, 12, 24
	12 a 20 contracciones	02 (8,33)	8 ,14
	15 a 40 contracciones	05 (20,8)	3, 6, 7, 17, 21
	1ª y 2ª semanas: 15 a 20 / 3ª y 4ª semanas: 30 a 40 / 5ª y 6ª semanas: 40 a 50 / 7ª a 26ª semana: 50 a 60	01(4,17)	18
	10 contracciones largas / 10 contracciones cortas	01 (4,17)	22
	15 contracciones lentas / 20 contracciones rápidas	01 (4,17)	19
**Tiempo de contracción**	No mencionado	06 (25,0)	1, 2, 7, 11, 13, 16
	3 a 5 segundos	07 (29,2)	3, 4, 5, 14, 17, 21, 23
	5 a 10 segundos	02 (8,33)	8, 10
	Comience con 2 a 3 segundos y aumente 1 segundo por día hasta llegar a 10 segundos	02 (8,33)	6, 20
	1ª contracción: 1-2 segundos, alternados con la segunda contracción: 6-7 segundos	01 (4,17)	12
	Fibras rápidas/cortas/rítmicas: 1 segundo; fibras lentas/largas/sostenidas: 5 a 10 segundos	06 (25,0)	9, 15, 18, 19, 22, 24
**Tiempo de relajación**	No mencionado	13 (54,2)	1, 2, 7, 9, 10, 11, 12, 13, 16, 17, 21, 22
	5 segundos	02 (8,33)	4, 20
	Comience con 4 segundos de relajación y aumente 2 segundos por día hasta llegar a 20 segundos. Luego reinicie los ejercicios con 4 segundos	01 (4,20)	6
	6 a 10 segundos	04 (16,7)	3, 5, 14, 23
	10 a 20 segundos	01 (4,17)	8
	2 minutos después de una serie de contracciones lentas y rápidas	01 (4,17)	15
	Rítmico: 1 segundo / Sostenido: 10 segundos	01 (4,17)	18
	Fibras lentas: 15 segundos y aumentar 1 segundo por semana; fibras rápidas: 10 segundos	01 (4,17)	19
**Intensidad de las contracciones**	No mencionado	22 (91,7)	1, 2, 4, 5, 6, 7, 8, 9, 11, 12, 13, 14, 15, 16, 17, 18, 10, 20, 21, 22, 23, 24
	Máximo (entrenamiento); submáximo (durante las actividades)	01 (4,17)	10
	70% de fuerza submáxima	01 (4,17)	3
**Posición de entrenamiento**	No mencionado	05 (24,8)	2, 5, 10, 13, 20
	Cualquier posición	01 (4,17)	21
	Posición supina	03 (12,5)	8, 16, 17
	Posición supina, sentada y de pie	09 (37,5)	1, 6, 9, 11, 18, 19, 22, 23, 24
	Posición supina, sentada y de pie, así como antes de cualquier esfuerzo o actividad física que pueda inducir incontinencia	05 (20,8)	3, 4, 12, 14, 15
	Inicialmente en decúbito lateral y, luego de acostumbrarse, en posición sentada y de pie	01 (4,17)	7
Músculo objetivo de la contracción*	No mencionado	11 (45,8)	1, 7, 9, 11, 12, 13, 15, 17, 20, 23, 24
	Esfínter anal/elevador del ano	05 (20,8)	3, 4, 6, 8, 14
	Esfínter uretral estriado	05 (20,8)	2, 16, 19, 21, 22
	Músculo bulbocavernoso	04 (16,7)	5, 8, 10, 18
**Cómo se proporcionó la información al paciente**	No mencionado	01 (4,17)	5
	Oral	08 (33,3)	1, 3, 6, 7, 16, 17, 23, 24
	Escrita	04 (16,7)	4, 11, 13, 21
	Oral y escrita	11 (45,8)	2, 8, 9, 10, 12, 14, 15, 19, 20, 22
**Profesionales que orientaron la realización del EMPP** ^†^	No mencionado	04 (16,7)	4, 5, 6, 19
	Fisioterapeuta	10 (41,7)	1, 3, 10, 11, 13, 14, 16, 17, 18, 22
	Urólogo	04 (16,7)	7, 12, 15, 23
	Enfermera	04 (16,7)	2, 9, 20, 21
	Enfermero o fisioterapeuta	01 (4,17)	8
	Enfermero o urólogo	01 (4,17)	24

* Un único estudio puede presentar más de un área como objetivo de entrenamiento;

^†^ EMPP = Entrenamiento de los músculos del piso pélvico

## Discusión

Los protocolos mapeados presentaron variaciones sustanciales en términos de contenido y diversidad de definiciones de continencia urinaria.

 La variabilidad en la descripción y la falta de consenso dificultan comprender cuándo comenzar el tratamiento, establecer la duración del tratamiento, definir el tiempo de contracción y relajación, entre otras pautas importantes para recuperar la continencia ^(^ 2022 ^,^ 2017 ^)^ . Al igual que en el presente estudio, una revisión sistemática de los protocolos para el tratamiento de los síntomas del tracto urinario inferior en hombres confirmó que había variabilidad en la información y faltaban descripciones de los ítems en los protocolos del EMPP ^(^ 2018 ^)^ . 

 La falta de detalles puede obedecer a que no se reconoce la importancia de dicha información o al límite de palabras impuesto por las revistas ^(^ 2018 ^)^ . La falta de descripción impide que se pueda replicar el estudio y realizar un análisis para evaluar la efectividad del protocolo. Una alternativa recomendable sería utilizar material complementario para describir el contenido, como apéndices o incluso la publicación del protocolo completo, tal como lo presentan los autores ^(^ 2009 ^)^ . 

 La diversidad de definiciones presentadas para el fenómeno de la IU hace que sea difícil definir el tiempo de recuperación de la continencia urinaria. La estandarización es necesaria para garantizar que se puedan comparar los resultados de los estudios ^(^ 2021 ^,^ 2021 ^)^ . 

 En lo que respecta al profesional responsable de realizar los protocolos de EMPP, se observa que prevaleció el abordaje de los fisioterapeutas. Sin embargo, en Brasil, otros profesionales pueden aplicar el EMPP, como enfermeros generales o especialistas en estomaterapia y/o urología, que cuentan con el apoyo del Consejo Federal de Enfermería y son considerados aptos para llevar a cabo terapias conservadoras para el tratamiento de la incontinencia urinaria post prostatectomía ^(^ 2016 ^)^ . 

 La mayoría de los estudios establecieron que era necesario implementar protocolos que duraran más de tres meses; sin embargo, una duración más corta se consideró efectiva para mejorar la IU en hombres, en términos de los efectos fisiológicos del EMPP ^(^ 2019 ^,^ 2022 ^)^ . Los efectos se pueden notar a las dos semanas, pero los síntomas disminuyen más significativamente entre las seis y ocho semanas de tratamiento ^(^ 2021 ^)^ . 

 Si bien se encontraron diversos protocolos, coincidían en el principio de contraer regularmente los músculos del piso pélvico para aumentar la presión de cierre de la uretra y, por ende, prevenir la pérdida de orina. Las pautas más citadas fueron realizar tres sesiones diarias de EMPP y mantener un intervalo de seis a 15 contracciones por sesión. Realizar de 50 a 60 contracciones por sesión entre la semana 7 y 26 de seguimiento ^(^ 2014 ^)^ dará como resultado un total de 180 contracciones por día. 

 En los protocolos también se encontró información sobre la progresión de la dificultad de los ejercicios a lo largo de las semanas, en función del cambio de posición, desde el nivel más fácil (acostado), al nivel intermedio (sentado) y al más difícil (de pie y durante las actividades diarias). Se sabe que, para fortalecer el piso pélvico, los hombres sometidos a prostatectomía radical deben poder ejercitar sus músculos contra la gravedad para soportar el contenido abdominal y prevenir la pérdida urinaria ^(^ 2021 ^)^ . 

 Esto es importante, dado que la prostatectomía radical no sólo afecta a la uretra y la próstata, sino también a la vejiga, desplazándola a un nivel inferior al que tenía originalmente antes de la cirugía ^(^ 2022 ^)^ . A medida que los pacientes se vuelven más fuertes, los músculos pueden ser sometidos a una carga mayor y a fuerzas gravitacionales que se oponen a la elevación del piso pélvico ^(^ 2009 ^)^ . 

 La progresión de las etapas del EMPP también puede incluir aumentar el tiempo de contracción y disminuir el tiempo de relajación hasta alcanzar el número definido por el protocolo, con el fin de evitar la fatiga temprana ^(^ 2018 ^,^ 2021 ^)^ . El tiempo medio de fatiga de los músculos del piso pélvico en mujeres con IU es de 11,5 segundos ^(^ 2004 ^)^ . Aunque hay que tener precaución al considerar la evidencia del EMPP en mujeres para los hombres, se cree que los períodos de contracción superiores a 10 segundos pueden causar fatiga temprana en algunos hombres ^(^ 2018 ^)^ . 

 Por otro lado, cabe señalar que el tiempo de contracción se asocia con el aumento de la fuerza y resistencia muscular ^(^ 2021 ^)^ . Por lo tanto, es importante que los pacientes aumenten el tiempo de contracción dentro de su capacidad para mejorar la resistencia de la uretra ^(^ 2020 ^,^ 2021 ^)^ . Por ende, el proceso de rehabilitación del piso pélvico implica la evaluación clínica individual de cada paciente, ya que, al identificar el reconocimiento adecuado de los músculos a ejercitar y su rendimiento ideal, se recomendarán ejercicios con mayor nivel de dificultad. 

 Otra pauta importante que se encuentra en los protocolos es realizar una contracción antes de aumentar la presión intraabdominal. Es fundamental que los pacientes se familiaricen con la forma adecuada de realizar los ejercicios, ya que pueden trabajar otros músculos irrelevantes, como los glúteos y los abdominales ^(^ 2022 ^)^ . 

 Al recomendar el EMPP, los profesionales deben explicarles a los pacientes cómo es la anatomía y cuál es la función de los músculos del piso pélvico ^(^ 2020 ^)^ , para que puedan reconocer los músculos que van a trabajar y realizar el entrenamiento propuesto. Sin embargo, la mayoría de los estudios mapeados no presentaron esta información. 

 Hay profesionales que asumen erróneamente que los pacientes contraerán correctamente los músculos del piso pélvico solamente con recibir instrucciones orales o materiales escritas. Sin embargo, el uso aislado de estas estrategias sin un seguimiento presencial puede conducir a la mala ejecución de los ejercicios ^(^ 2021 ^,^ 2023 ^)^ . 

 Otro punto a considerar es que la mayoría de los estudios no declaran ni hacen referencia a la fuente de las instrucciones que utilizaron en sus intervenciones. Varios protocolos aplican a los hombres principios que fueron creados para mujeres con IU por esfuerzo, lo que puede comprometer la eficacia de la intervención ^(^ 2020 ^,^ 2018 ^,^ 2021 ^)^ , debido a las diferencias anatómicas y las variaciones del mecanismo de la IU ^(^ 2020 ^)^ . 

 A diferencia de los hombres, en las mujeres el mecanismo común de la IU es la disfunción de los músculos elevadores del ano, que es un efecto secundario del embarazo y del parto vaginal ^(^ 2011 ^-^ 2008 ^)^ . Por lo tanto, el EMPP en hombres debe considerar la fisiopatología de la incontinencia urinaria post prostatectomía, especialmente en lo que respecta al aumento de la activación del esfínter uretral estriado para compensar la pérdida/reducción del esfínter interno y al mantenimiento de la distensibilidad de la vejiga para reducir la hiperactividad del detrusor ^(^ 2020 ^,^ 2021 ^)^ . 

 A pesar de la evidencia de que el objetivo del EMPP en los hombres debería ser el esfínter uretral estriado ^(^ 2020 ^,^ 2018 ^,^ 2021 ^)^ , algunos estudios se centran en el esfínter anal y el músculo bulbocavernoso. Esas instrucciones pueden comprometer el efecto del tratamiento, ya que la contracción aislada alrededor del ano no activa los músculos que contraen la uretra ^(^ 2020 ^,^ 2021 ^)^ . 

 La información para identificar correctamente los músculos se basa en intentar controlar la micción sin contraer los músculos abdominales, los glúteos y la parte interna del muslo. Sin embargo, la participación de los músculos abdominales para fortalecer los músculos pélvicos no está clara en la literatura, dado que existen hipótesis de que la baja efectividad del EMPP, en algunos casos, está asociada con la subutilización de los músculos abdominales, lo que puede limitar la actividad del músculo pubococcígeo. Este hecho justifica el efecto de otros tratamientos conservadores, como yoga y pilates ^(^ 2020 ^)^ , pero los protocolos encontrados en esta revisión no recomiendan el uso de esos músculos. 

Debido a la especificidad y el detalle de las medidas descritas en los protocolos del EMPP, es evidente la importancia que tiene el seguimiento profesional presencial y la aclaración con información escrita de fácil comprensión, ya sea de forma impresa o digital. Además, para que el plan terapéutico sea efectivo, siempre hay que combinarlo con otras intervenciones conductuales imprescindibles para el control paulatino de la continencia, como los hábitos alimentarios, la actividad física y el uso del baño.

Aunque el 80% de los protocolos de EMPP incluidos en la muestra se obtuvieron de estudios controlados y aleatorizados, la heterogeneidad metodológica de los estudios limitó la posibilidad de comparar resultados.

Sin embargo, los hallazgos de esta revisión presentan importantes implicaciones clínicas y para la investigación que respaldan el sentido crítico de los profesionales de la salud en la toma de decisiones.

Los aspectos destacados en los protocolos, como la intensidad de las contracciones, el número de sesiones, el tiempo de relajación y las contracciones sostenidas, pueden ser de gran utilidad para el manejo de casos específicos en la práctica clínica. Además, la síntesis de información de los protocolos incluidos en este estudio puede contribuir significativamente a la enseñanza clínica de estas prácticas en las carreras de grado y posgrado del área de la salud. Esto promueve el sentido crítico de los profesionales, les permite tomar decisiones más asertivas y fomenta la práctica basada en evidencia. La inclusión de estos hallazgos en la formación de los enfermeros promueve la aplicación de la mejor evidencia científica disponible en la toma de decisiones clínicas y garantiza intervenciones más efectivas y seguras.

## Conclusión

Esta revisión destacó que el EMPP es una intervención convencional eficaz, económica y no invasiva de primera elección para el tratamiento de la incontinencia urinaria post prostatectomía, que hay que comenzar inmediatamente después de retirar el catéter urinario permanente.

También proporcionó una visión general de los aspectos incluidos en los protocolos de EMPP en hombres con incontinencia urinaria post prostatectomía, pero señaló que faltan estudios que describan los protocolos en detalle. Por lo tanto, es cuestionable el consenso sobre cuáles son las instrucciones que hay que seguir con respecto al inicio del EMPP, la posición, el tiempo de contracción/relajación, la intensidad de la contracción y la duración de los protocolos.

En vista de esta falta de detalles, se recomienda realizar una observación clínica cuidadosa y un informe adecuado del método y los resultados de cualquier propuesta futura en la búsqueda de la mejor evidencia.

Para avanzar en las discusiones y prácticas relacionadas con la incontinencia urinaria post prostatectomía, es necesario desarrollar guías consensuadas que definan claramente los parámetros de los protocolos de entrenamiento. Los resultados obtenidos del mapeo realizado pueden contribuir a crear una lista que reúna las prácticas más citadas en la literatura, cuya eficacia se pueda evaluar en futuros ensayos clínicos, con el objetivo de estandarizar un protocolo de entrenamiento de la musculatura del piso pélvico.

# Translation: Treinamento dos músculos do assoalho pélvico em homens com incontinência urinária pós-prostatectomia: revisão do escopo*

**DOI:** 10.1590/1518-8345.7335.4387

**Published:** 2024-10-04

**Authors:** Jackelline Evellin Moreira dos Santos, Virginia Visconde Brasil, Cissa Azevedo, Lívia Cristina de Resende Izidoro, Anna Julia Guimarães Batista, André Carlos Santos Ferreira, Luciana Regina Ferreira da Mata

**Affiliations:** 1 Universidade Federal de Goiás, Faculdade de Enfermagem, Goiânia, GO, Brasil.; ^2^ Universidade Federal de São João del Rei, Curso de Enfermagem, Divinópolis, MG, Brasil.; ^3^ Universidade Federal de Jataí, Curso de Enfermagem, Jataí, GO, Brasil.; ^4^ Universidade Federal de Minas Gerais, Escola de Enfermagem, Belo Horizonte, MG, Brasil.

**Keywords:** Prostatectomia, Incontinência Urinária, Terapia Cognitivo-Comportamental, Diafragma Pélvico, Tratamento Conservador, Terapia por Exercício

## Abstract

mapear os protocolos de treinamento dos músculos do assoalho pélvico disponíveis na literatura para o tratamento da incontinência urinária pós-prostatectomia.

trata-se de uma revisão de escopo realizada em seis bases de dados. A recuperação de informações foi realizada por meio de um instrumento específico, incluindo: título, autoria, ano de publicação, periódico, objetivo, desenho do estudo e descrição do conteúdo dos protocolos.

um total de 24 estudos foram incluídos, o que resultou em diferentes protocolos. As recomendações mais frequentes foram as seguintes: três sessões por dia; com frequência de seis a 15 contrações por sessão; realização dos exercícios nas posições deitada, sentada e em pé. As diretrizes mais citadas nos protocolos foram as contrações do esfíncter anal e do músculo bulbocavernoso. Todos os protocolos seguiram o princípio da contração regular dos músculos do assoalho pélvico, mas não houve consenso quanto ao início do protocolo, duração do tratamento, tempo de contração/relaxamento e intensidade da força de contração.

foram encontradas diferentes orientações nos protocolos de treinamento dos músculos do assoalho pélvico para incontinência urinária pós-prostatectomia, com ênfase no tempo de contração, tempo de relaxamento, número de contrações por sessão, posição de treinamento e tempo de duração do protocolo. Diferentes conceitos foram adotados para definir a continência urinária nos protocolos. É necessário desenvolver diretrizes de consenso que definam claramente os parâmetros dos protocolos de treinamento.

## Destaques

**(1)** O treinamento dos músculos do assoalho pélvico é uma intervenção eficaz de primeira escolha.

**(2)** Escassez de estudos que descrevam detalhadamente os protocolos de treinamento dos músculos do assoalho pélvico.

**(3)** Não há consenso entre as instruções contidas nos diferentes protocolos

## Introdução

 A incontinência urinária (IU) é definida como a queixa de qualquer perda involuntária de urina ^(^ 2022 ^)^ , sendo uma complicação comum em homens submetidos à prostatectomia radical, independentemente do tipo de procedimento cirúrgico ^(^ 2020 ^)^ . 

 Embora ainda não totalmente compreendida, a etiologia da incontinência urinária pós-prostatectomia está relacionada à diminuição ou perda da integridade, força e/ou ativação da musculatura do assoalho pélvico ^(^ 2021 ^-^ 2021 ^)^ . Entre os principais fatores que afetam a continência pós-operatória, podem ser mencionados aqueles relacionados ao paciente (idade, obesidade e índice de massa corporal), biológicos (disfunção urinária pré-operatória, formato e tamanho da próstata e complacência uretral) e cirúrgicos (preservação do colo vesical ou reconstrução das estruturas de suporte uretral) ^(^ 2021 ^-^ 2023 ^)^ . Assim, são necessárias avaliações clínicas específicas para direcionar o tipo de tratamento mais eficaz de acordo com sua origem etiológica ^(^ 2021 ^)^ . 

 A incontinência urinária pós-prostatectomia tem ampla variação na prevalência (2% a 60%), dependendo da metodologia usada para sua avaliação ^(^ 2020 ^)^ . Pode ser uma condição transitória, com recuperação dentro de seis meses após a prostatectomia radical. Em alguns casos, os sintomas persistem 12 meses após a cirurgia ^(^ 2019 ^)^ , o que tem um impacto significativo no bem-estar psicológico e na qualidade de vida desses indivíduos ^(^ 2020 ^,^ 2022 ^)^ . 

 Em relação ao manejo terapêutico, é aconselhável realizar o manejo inicial o mais cedo possível ^(^ 2019 ^)^ , de forma conservadora. Essa modalidade inclui, entre outras intervenções, exercícios para os músculos pélvicos ^(^ 2020 ^)^ , apresentados na literatura com o termo “treinamento dos músculos do assoalho pélvico” ^(^ 2023 ^)^ , isolados ou combinados com estimulação elétrica e/ou *biofeedback* ^(^ 2018 ^)^ . 

 O treinamento dos músculos do assoalho pélvico (TMAP) consiste em contrações repetidas dos músculos pélvicos para melhorar a força, a resistência e a coordenação muscular ^(^ 2020 ^,^ 2022 ^)^ . Alguns dos principais músculos envolvidos na continência urinária masculina no TMAP são o esfíncter uretral externo, o levantador do ânus e o bulbocavernoso, que são ativados em momentos diferentes de coordenação ^(^ 2020 ^)^ . 

 Nenhum protocolo padronizado para a realização do TMAP nessa população foi estabelecido na literatura. No entanto, a *International Continence Society* (Sociedade Internacional de Continência) ^(^ 2023 ^)^ recomenda o TMAP como o tratamento conservador de primeira escolha, devido aos benefícios significativos para o estado de continência e ao impacto reduzido na qualidade de vida de homens com incontinência urinária pós-prostatectomia ^(^ 2020 ^,^ 2018 ^)^ . 

 Sabe-se que a tomada de decisão sobre a melhor conduta que garanta a prática assistencial inclui a revisão das evidências disponíveis na literatura. No entanto, a variabilidade das diretrizes fornecidas aos homens para a conscientização e propriocepção muscular, o programa de exercícios e a forma de avaliação para verificar a ativação correta dos músculos do assoalho pélvico dificultam a definição de protocolos eficazes na prática clínica ^(^ 2020 ^,^ 2019 ^)^ . 

 Vale ressaltar que se trata de uma intervenção de baixo custo, baixo risco, minimamente invasiva, com contraindicações mínimas ^(^ 2017 ^)^ e com evidências de eficácia para o tratamento da incontinência urinária pós-prostatectomia ^(^ 2020 ^)^ . Nesse contexto, considerando a importância do conhecimento dos protocolos do TMAP para garantir a recuperação da continência, justifica-se este estudo para sintetizar o escopo do conteúdo encontrado nos programas. 

O objetivo deste estudo foi mapear os protocolos de exercícios para os músculos pélvicos disponíveis na literatura para o tratamento da incontinência urinária pós-prostatectomia.

## Método

### Desenho do estudo

 Esta é uma revisão de escopo conduzida de acordo com as revisões de escopo do *Joanna Briggs Institute* ^(^ 2020 ^)^ e relatada de acordo com a lista de verificação *Preferred Reporting Items for Systematic reviews and Meta-Analyses extension for Scoping Reviews* (PRISMA-ScR) ^(^ 2018 ^)^ , nas seguintes etapas: definição e alocação de objetivos e perguntas de pesquisa; preparação de critérios de inclusão; concepção e planejamento da estratégia de busca e seleção de estudos; identificação e seleção de estudos relevantes; extração e mapeamento de dados; e resumo dos resultados. O protocolo de revisão foi registrado no Open Science Framework com o número de registro DOI 10.17605/OSF.IO/HC4ZX. 

### Identificação da pergunta de pesquisa

Para orientar a busca, a pergunta de pesquisa foi preparada seguindo a estratégia PCC (População-Conceito-Contexto), em que P - homens com incontinência urinária; C - protocolos do TMAP para o tratamento da incontinência urinária; C - cirurgia de prostatectomia pós-radical: “Quais são os protocolos de treinamento dos músculos do assoalho pélvico descritos na literatura para o tratamento da incontinência urinária pós-prostatectomia?”.

### Critérios de seleção

 Foram incluídos estudos primários completos e originais disponíveis online em periódicos nacionais e internacionais, em português, inglês e espanhol, independentemente do ano de publicação, que apresentassem diretrizes/protocolos relacionados ao TMAP para homens com incontinência urinária pós-prostatectomia. Foram incluídos estudos que tinham como objetivo avaliar o efeito do *biofeedback* ou da eletroestimulação combinados com o TMAP, desde que apresentassem pelo menos um grupo submetido a um protocolo isolado do TMAP. 

 Foram excluídos editoriais, cartas de resposta, estudos secundários, relatos de experiência ou opinião de especialistas; intervenções de reabilitação tardia (início do tratamento após um ano da prostatectomia radical); protocolos do TMAP combinados com eletroestimulação, *biofeedback* , vibração e estimulação magnética; TMAP combinados com tratamento farmacológico e/ou cirúrgico; publicações com o mesmo protocolo de estudo; relatos e séries de casos; e literatura cinzenta. 

### Estratégia de pesquisa

 Um método de três etapas foi usado para desenvolver a estratégia de pesquisa ^(^ 2020 ^)^ : 1. Pesquisa realizada em dois bancos de dados para recuperar termos MeSH e palavras-chave; 2. Pesquisa bibliográfica realizada em bancos de dados virtuais; 3. Pesquisa bibliográfica de listas de referências. 

 A primeira etapa incluiu pesquisa no *Medical Literature Analysis and Retrieval System Online* (MEDLINE), por meio da *National Library of Medicine* (PubMed), e nos bancos de dados do *site* da Biblioteca Virtual em Saúde (BVS), para verificar os principais descritores ou palavras-chave usados nos estudos relacionados à questão norteadora. 

 Os termos controlados selecionados foram verificados nos Descritores em Ciências da Saúde (DeCS) e no *Medical Subject Heading* (MeSH): *Prostatectomy* , *Urinary Incontinence* , *Behavior Therapy* , *Pelvic Floor* , *Muscle* , *Exercise Therapy* , *Conservative Treatment* , *Rehabilitation* , *Training Support* , *Contraction* , *Education and Lifestyle* , e *Healthy lifestyle* . 

 A segunda etapa foi realizada em 5 de maio de 2022, nos seguintes bancos de dados: MEDLINE, PubMed; Biblioteca *Cochrane* ; SCOPUS, *Biomedical Answer* (EMBASE); no *site* da BVS e na *Web of Science* (WoS). 

 Decidiu-se usar descritores controlados (DeCS e MesH) e palavras-chave (descritores não controlados) em português, inglês e espanhol, a fim de obter estratégia de pesquisa direcionada. Foi delineada estratégia única, que foi adaptada para cada banco de dados listado. Foram utilizados os operadores booleanos AND e OR, conforme mostrado na Figura 1 . 

**Figura 1:** - Estratégias de pesquisa aplicadas e adaptadas a cada banco de dados. Goiânia, GO, Brasil, 2022

### Processo de seleção do estudo

 O gerenciador de referências *EndNote* (versão X9 - *Desktop* ) foi usado para remover duplicatas dos resultados exportados dos seis bancos de dados. A plataforma *Rayyan* foi usada para auxiliar no processo de organização e seleção dos estudos primários pelos revisores ^(^ 2016 ^)^ . A seleção incluiu a leitura dos títulos e resumos das publicações, com base na pergunta norteadora e nos critérios de elegibilidade. Essa etapa foi realizada por dois revisores de forma independente e cega. O cegamento da plataforma *Rayyan* foi aberto e, em reuniões de consenso, os revisores selecionaram os estudos para leitura completa, com a participação de um terceiro revisor. Os estudos foram lidos na íntegra por dois revisores de forma independente e, em casos de discordância, um terceiro revisor foi consultado. 

A terceira etapa da pesquisa foi realizada pesquisando manualmente as referências dos estudos secundários não incluídos para identificar estudos que atendessem aos critérios de seleção e que não tivessem sido identificados anteriormente.

### Instrumentos usados para coletar informações

 Para coletar e categorizar as informações, foi utilizada ferramenta de extração de dados baseada na literatura ^(^ 2020 ^,^ 2018 ^,^ 2017 ^)^ . O instrumento foi refinado por três pesquisadores de enfermagem com experiência nesse campo e incluiu os seguintes itens: título da publicação; autor(es); ano da publicação; periódico; objetivo; desenho do estudo; conceito adotado para continência e descrição do conteúdo dos protocolos do TMAP. 

### Tratamento e análise de dados

Após a categorização, os dados foram sintetizados para análise descritiva de acordo com o ano de publicação, o objetivo, o idioma, o tipo de estudo, o conceito de continência e os itens do protocolo do TMAP (horário de início do tratamento, duração do protocolo, número de sessões por dia, número de contrações por sessão, tempo de contração, tempo de relaxamento, posições do TMAP, músculo alvo da contração e a forma como as informações foram fornecidas).

### Aspectos éticos

Como se tratava de análise de escopo, a pesquisa não foi submetida à consideração de um Comitê de Ética em Pesquisa.

## Resultados

 Foi encontrado um total de 2.163 artigos na literatura. Após a análise do título e do resumo, 114 artigos foram lidos na íntegra e 24 foram incluídos na revisão ( Figura 2 ). 

 Os estudos foram publicados entre 2000 e 2022, sendo que nove (37,5%) foram publicados na última década. O idioma predominante foi o inglês (n=22; 91,7%). Os estudos foram realizados em 15 países diferentes, sendo que a Itália (n=4; 16,7%) teve o maior número de publicações. Quanto ao tipo de estudo, 21 (87,5%) foram classificados como ensaios clínicos randomizados, publicados em 18 periódicos diferentes ( Figura 3 ). 

 Dos 24 protocolos de PFMT mapeados, quatro (16,7%) forneceram descrição do conteúdo completo dos protocolos ^(^ 2004 ^-^ 2008 ^)^ . Três estudos (12,5%) abordaram ou fizeram referência à origem das instruções apresentadas no protocolo ^(^ 2009 ^,^ 2007 ^-^ 2019 ^)^ . 

 Em relação à definição de continência urinária, 10 (41,7%) estudos não forneceram a conceituação ^(^ 2004 ^,^ 2009 ^,^ 2007 ^,^ 2014 ^-^ 2010 ^)^ ; três (12,5%) consideraram continência como a ausência de uso de absorventes ^(^ 2008 ^-^ 2021 ^)^ ; dois (8,33%) consideraram como uso de até um absorvente por dia; um (4,17%) como perda de urina < 10 g ^(^ 2019 ^)^ ; um (4,17%) como perda de urina ≤ 8 g em 24 horas ^(^ 2008 ^)^ ; dois (8,33%) como perda de urina < 1 g no teste de absorvente de uma hora ^(^ 2002 ^,^ 2010 ^)^ ; quatro (16,7%) como perda de urina ≤ 2 g no teste de absorvente de 24 horas ^(^ 2007 ^-^ 2000 ^)^ ; e um (4,17%) como nenhuma perda de urina no teste de absorvente de 24 horas ^(^ 2020 ^)^ . 

**Figura 2:** - Fluxograma do processo de seleção dos estudos incluídos na revisão de escopo, adaptado do PRISMA *Extension for Scoping Reviews* (PRISMA-ScR) ^(^ 2018 ^)^ . Goiânia, GO, Brasil, 2022

**Figura 3:** - Classificação dos estudos incluídos no mapeamento dos protocolos de treinamento dos músculos do assoalho pélvico (n = 24). Goiânia, GO, Brasil, 2022

*TMAP = Treinamento dos músculos do assoalho pélvico

 As informações mais comuns relatadas pelos protocolos foram as seguintes: número de sessões por dia (n=22; 91,7%); tempo de duração do protocolo (n=19; 79,2%); posição de treinamento (n=19; 79,2%); horário de início do tratamento (n=18; 75%); tempo de contração (n=18; 75%); e número de contrações por sessão (n=17; 70,8%). As informações menos descritas foram as seguintes: intensidade das contrações (n=22; 91,7%); músculo exercitado durante a contração (n=12; 50%) e tempo de relaxamento (n=11; 45,8%) ( Tabela 1 ). 

 A maioria das informações foi fornecida aos pacientes na forma falada e escrita (n=11; 45,8%), ou somente na forma falada (n=8; 33,3%) ( Tabela 1 ). Dos protocolos analisados, oito (33,3%) mencionaram a necessidade de instruções sobre a anatomia e/ou fisiologia do assoalho pélvico e sobre os mecanismos relacionados à IU ^(^ 2002 ^-^ 2009 ^,^ 2014 ^,^ 2010 ^-^ 2008 ^,^ 2010 ^,^ 2000 ^-^ 2020 ^)^ . 

 Com relação ao tempo de início do protocolo, 13 (54,2%) estudos recomendam o início do TMAP até 15 dias após a prostatectomia radical. A duração mais frequente do TMAP nos estudos foi de até seis meses (n=8; 33,3%), com um tempo mínimo de 10 semanas (n=1; 4,17%) e um máximo de 12 meses (n=5; 20,8%). Com relação ao número de sessões diárias, a maioria dos protocolos relatou até três sessões diárias (n=18; 75%), em três períodos do dia (manhã, tarde e noite) ( Tabela 1 ). 

 O número de contrações por sessão foi um dos aspectos mais diversos na literatura; sete (29,2%) protocolos relataram de seis a 15 contrações, e cinco protocolos (20,8%) relataram de 15 a 40 contrações. Dois (8,33%) estudos sugeriram aumentar o número de contrações à medida que o tratamento progredia ( Tabela 1 ). 

 Quanto ao tempo de contração, sete (29,2%) protocolos recomendaram de três a cinco segundos. Quanto ao tempo de relaxamento, quatro (16,7%) protocolos indicaram de seis a 10 segundos, sendo que o maior tempo recomendado foi de 20 segundos (n=2; 8,33%) ( Tabela 1 ). 

 Em relação à posição de realização do TMAP, nove (37,5%) protocolos mencionaram a posição supina, sentada e em pé. A orientação de realizar o TMAP antes de qualquer esforço ou atividade que possa induzir a IU foi mencionada por cinco (20,8%) protocolos ( Tabela 1 ). 

 No que se refere aos músculos exercitados durante o TMAP, cinco (20,8%) protocolos declararam que se concentraram no músculo elevador do ânus e quatro (12,5%) no músculo bulbocavernoso ( Tabela 1 ). 

**Tabela 1:** - Frequência dos itens apresentados no mapeamento dos protocolos de treinamento dos músculos do assoalho pélvico (n = 24). Goiânia, GO, Brasil, 2023

**Itens do protocolo**	**Detalhamento dos itens do protocolo**	**Frequência** **n (%)**	**Estudos realizados**
**Momento em que o treinamento começa**	Não mencionado	06 (25,0)	6, 7, 9, 16, 17, 21
	Até 15 dias após a prostatectomia radical	13 (54,2)	1, 2, 3, 4, 11, 13, 14, 15, 18, 20, 22, 23, 24
	Até 30 dias após a prostatectomia radical	02 (8,33)	7, 8
	> 30 dias após a prostatectomia radical	03 (12,5)	10, 12, 19
**Duração do protocolo**	Não mencionado	05 (20,8)	1, 9, 11, 16, 19
	Até 3 meses	06 (25,0)	4, 10, 14, 20, 21,22
	Até 6 meses	08 (33,3)	2, 3, 5, 13, 15, 17, 18, 23
	> 6 meses	05 (20,8)	6, 7, 8, 12, 24
**Sessões por dia**	Não mencionado	02 (8,33)	1, 23
	Até 3 sessões	18 (75,0)	5, 6, 7, 8, 9, 10, 12, 13, 14, 15, 16, 17, 18, 19, 20, 21, 22, 24
	> de 3 sessões	04 (16,7)	2, 3, 4, 11
**Contrações por sessão**	Não mencionado	07 (29,2)	1, 5, 13, 15, 16, 20, 23
	06 a 15 contrações	07 (29,2)	2, 4, 9, 10, 11, 12, 24
	12 a 20 contrações	02 (8,33)	8 ,14
	15 a 40 contrações	05 (20,8)	3, 6, 7, 17, 21
	1ª e 2ª semanas: 15 a 20 / 3ª e 4ª semanas: 30 a 40 / 5ª e 6ª semanas: 40 a 50 / 7ª a 26ª semana: 50 a 60	01(4,17)	18
	10 contrações longas / 10 contrações curtas	01 (4,17)	22
	15 contrações lentas / 20 contrações rápidas	01 (4,17)	19
**Tempo de contração**	Não mencionado	06 (25,0)	1, 2, 7, 11, 13, 16
	3 a 5 segundos	07 (29,2)	3, 4, 5, 14, 17, 21, 23
	5 a 10 segundos	02 (8,33)	8, 10
	Começa de 2 a 3 segundos e aumenta em 1 segundo por dia até chegar a 10 segundos	02 (8,33)	6, 20
	1ª contração: 1-2 segundos, alternados com a segunda contração: 6-7 segundos	01 (4,17)	12
	Fibras rápidas/curtas/rítmicas: 1 segundo; fibras lentas/longas/sustentadas: 5 a 10 segundos	06 (25,0)	9, 15, 18, 19, 22, 24
**Tempo de relaxamento**	Não mencionado	13 (54,2)	1, 2, 7, 9, 10, 11, 12, 13, 16, 17, 21, 22
	5 segundos	02 (8,33)	4, 20
	Comece com 4 segundos de relaxamento e aumente 2 segundos por dia até chegar a 20 segundos. Em seguida, reinicie os exercícios com 4 segundos	01 (4,20)	6
	6 a 10 segundos	04 (16,7)	3, 5, 14, 23
	10 a 20 segundos	01 (4,17)	8
	2 minutos após uma série de contrações lentas e rápidas	01 (4,17)	15
	Rítmico: 1 segundo / Sustentado: 10 segundos	01 (4,17)	18
	Fibras lentas: 15 segundos e aumentar em 1 segundo a cada semana; fibras rápidas: 10 segundos	01 (4,17)	19
**Intensidade das contrações**	Não mencionado	22 (91,7)	1, 2, 4, 5, 6, 7, 8, 9, 11, 12, 13, 14, 15, 16, 17, 18, 10, 20, 21, 22, 23, 24
	Máximo (treinamento); submáximo (durante as atividades)	01 (4,17)	10
	70% de força submáxima	01 (4,17)	3
**Posição de treinamento**	Não mencionado	05 (24,8)	2, 5, 10, 13, 20
	Qualquer posição	01 (4,17)	21
	Posição supina	03 (12,5)	8, 16, 17
	Posição supina, sentada e em pé	09 (37,5)	1, 6, 9, 11, 18, 19, 22, 23, 24
	Posição supina, sentada e em pé, bem como antes de qualquer esforço físico ou atividade que possa induzir a incontinência	05 (20,8)	3, 4, 12, 14, 15
	Inicialmente em decúbito lateral e, depois de se acostumar, nas posições sentada e em pé	01 (4,17)	7
Músculo visado para contração*	Não mencionado	11 (45,8)	1, 7, 9, 11, 12, 13, 15, 17, 20, 23, 24
	Esfíncter anal / elevador do ânus	05 (20,8)	3, 4, 6, 8, 14
	Esfíncter uretral estriado	05 (20,8)	2, 16, 19, 21, 22
	Músculo bulbocavernoso	04 (16,7)	5, 8, 10, 18
**Como as informações foram fornecidas ao paciente**	Não mencionado	01 (4,17)	5
	Falado	08 (33,3)	1, 3, 6, 7, 16, 17, 23, 24
	Escrito	04 (16,7)	4, 11, 13, 21
	Falado e escrito	11 (45,8)	2, 8, 9, 10, 12, 14, 15, 19, 20, 22
**Profissionais que orientaram o desempenho TMAP** ^†^	Não mencionado	04 (16,7)	4, 5, 6, 19
	Fisioterapeuta	10 (41,7)	1, 3, 10, 11, 13, 14, 16, 17, 18, 22
	Urologista	04 (16,7)	7, 12, 15, 23
	Enfermeira	04 (16,7)	2, 9, 20, 21
	Enfermeiro ou fisioterapeuta	01 (4,17)	8
	Enfermeiro ou urologista	01 (4,17)	24

* Um único estudo pode apresentar mais de uma área como alvo de treinamento

^†^ TMAP = Treinamento dos músculos do assoalho pélvico

## Discussão

Os protocolos mapeados apresentaram variação substancial em termos de conteúdo e diversidade de definições de continência urinária.

 A variabilidade na descrição e a falta de consenso dificultam a compreensão de quando iniciar o tratamento, estabelecer a duração do tratamento, definir o tempo de contração e relaxamento, entre outras diretrizes importantes para a recuperação da continência ^(^ 2022 ^,^ 2017 ^)^ . Corroborando esses achados, revisão sistemática dos protocolos para o tratamento dos sintomas do trato urinário inferior em homens confirmou a variabilidade das informações e a falta de descrições dos itens nos protocolos do TMAP ^(^ 2018 ^)^ . 

 A falta de detalhes relatados pode ser explicada tanto pela falta de reconhecimento da relevância de tais informações quanto pelo limite de palavras imposto pelas revistas ^(^ 2018 ^)^ . A falta de descrição impede a replicação do estudo e a análise para avaliar a eficácia do protocolo. Como alternativa, seria aconselhável usar materiais complementares para descrever o conteúdo como apêndices ou até mesmo a publicação do protocolo na íntegra, conforme apresentado pelos autores ^(^ 2009 ^)^ . 

 A diversidade de definições apresentadas para o fenômeno da IU leva à dificuldade de definir o tempo para a recuperação da continência urinária. A padronização é necessária para garantir que os resultados dos estudos possam ser comparados ^(^ 2021 ^,^ 2021 ^)^ . 

 Em relação ao profissional responsável pela condução dos protocolos de TMAP, observou-se prevalência de abordagens realizadas por fisioterapeutas. Entretanto, no Brasil, outros profissionais podem aplicar o TMAP, como enfermeiros generalistas ou especialistas em estomaterapia e/ou urologia, que têm o respaldo do Conselho Federal de Enfermagem e são considerados aptos a conduzir terapias conservadoras para o tratamento da incontinência urinária pós-prostatectomia ^(^ 2016 ^)^ . 

 A maioria dos estudos estabeleceu a necessidade de protocolos que durassem mais de três meses; no entanto, duração mais curta foi considerada eficaz para melhorar a IU em homens, em termos dos efeitos fisiológicos do TMAP ^(^ 2019 ^,^ 2022 ^)^ . Os efeitos podem ser notados após duas semanas, embora os sintomas diminuam mais significativamente entre seis e oito semanas de tratamento ^(^ 2021 ^)^ . 

 Embora tenha sido encontrada variedade de protocolos, houve consenso sobre o princípio de contrair regularmente os músculos do assoalho pélvico para aumentar a pressão de fechamento da uretra e, consequentemente, evitar a perda de urina. As diretrizes mais citadas foram a realização de três sessões diárias de TMAP e a manutenção de intervalo de seis a 15 contrações por sessão. A realização de 50 a 60 contrações por sessão entre a 7ª e a 26ª semana de acompanhamento ^(^ 2014 ^)^ , resultará em um total de 180 contrações por dia. 

 Informações sobre a progressão da dificuldade dos exercícios ao longo das semanas também foram encontradas nos protocolos, a partir da mudança de posicionamento, do nível mais fácil (deitado), ao intermediário (sentado) e ao mais difícil (em pé e durante a realização das atividades de vida diária). Sabe-se que, para fortalecer o assoalho pélvico, os homens submetidos à prostatectomia radical devem se tornar capazes de exercitar os músculos contra a gravidade para sustentar o conteúdo abdominal e evitar a perda urinária ^(^ 2021 ^)^ . 

 Isso é relevante, pois a prostatectomia radical afeta não apenas a uretra e a próstata, mas também a bexiga, movendo-a para nível mais baixo do que o originalmente existente antes da cirurgia ^(^ 2022 ^)^ . À medida que os pacientes se tornam mais fortes, os músculos podem ser submetidos a carga maior e a forças gravitacionais que se opõem à elevação do assoalho pélvico ^(^ 2009 ^)^ . 

 A progressão das etapas do TMAP também pode incluir o aumento do tempo de contração e a diminuição do tempo de relaxamento até atingir o número definido pelo protocolo, a fim de evitar a fadiga precoce ^(^ 2018 ^,^ 2021 ^)^ . O tempo médio para a fadiga dos músculos do assoalho pélvico em mulheres com IU é de 11,5 segundos ^(^ 2004 ^)^ . Embora seja necessário ter cautela ao considerar as evidências de TMAP em mulheres para homens, acredita-se que períodos de contração superiores a 10 segundos possam causar fadiga precoce em alguns homens ^(^ 2018 ^)^ . 

 Por outro lado, vale a pena observar que o tempo de contração está associado a ganhos de força e resistência muscular ^(^ 2021 ^)^ . Portanto, é importante que os pacientes aumentem o tempo de contração dentro de sua capacidade para melhorar a resistência uretral ^(^ 2020 ^,^ 2021 ^)^ . Assim, o processo de reabilitação do assoalho pélvico implica a avaliação clínica individual de cada paciente, pois, ao identificar o reconhecimento adequado dos músculos a serem exercitados e o desempenho ideal, serão recomendados exercícios com maior nível de dificuldade. 

 Outra diretriz importante encontrada nos protocolos diz respeito à realização de contração antes do aumento da pressão intra-abdominal. É essencial que os pacientes se familiarizem com a maneira adequada de realizar os exercícios, pois outros músculos irrelevantes, como os músculos glúteos e abdominais, podem ser recrutados ^(^ 2022 ^)^ . 

 Ao recomendar o TMAP, os profissionais devem explicar a anatomia e a função dos músculos do assoalho pélvico aos pacientes ^(^ 2020 ^)^ , a fim de esclarecer o reconhecimento da musculatura para a execução adequada do treinamento proposto. Entretanto, a maioria dos estudos mapeados não apresentou essa informação. 

 Nesse contexto, há profissionais que erroneamente supõem que as pacientes contrairão corretamente os músculos do assoalho pélvico ao receberem apenas instruções faladas ou materiais escritos. Entretanto, o uso isolado dessas estratégias sem acompanhamento presencial pode levar a exercícios mal executados ^(^ 2021 ^,^ 2023 ^)^ . 

 Outro ponto a ser considerado é que a maioria dos estudos não declara ou faz referência à fonte das instruções usadas em suas intervenções. Vários protocolos aplicam princípios criados para mulheres com IU de esforço em homens, o que pode comprometer a eficácia da intervenção ^(^ 2020 ^,^ 2018 ^,^ 2021 ^)^ , considerando as diferenças anatômicas e as variações no mecanismo da IU ^(^ 2020 ^)^ . 

 Diferentemente dos homens, nas mulheres, o mecanismo comum da IU é a disfunção dos músculos elevadores do ânus, que é secundária à gravidez e ao parto vaginal ^(^ 2011 ^-^ 2008 ^)^ . Portanto, o TMAP em homens deve considerar a fisiopatologia da incontinência urinária pós-prostatectomia, especialmente no que se refere ao aumento da ativação do esfíncter uretral estriado para compensar a perda/redução do esfíncter interno e à manutenção da complacência da bexiga para reduzir a hiperatividade do detrusor ^(^ 2020 ^,^ 2021 ^)^ . 

 Apesar das evidências de que o alvo do TMAP em homens deva ser o esfíncter uretral estriado ^(^ 2020 ^,^ 2018 ^,^ 2021 ^)^ , alguns estudos têm como alvo o esfíncter anal e o músculo bulbocavernoso. Essas são instruções que podem comprometer o efeito do tratamento, pois a contração isolada ao redor do ânus não ativa os músculos que contraem a uretra ^(^ 2020 ^,^ 2021 ^)^ . 

 As informações para a identificação correta dos músculos baseiam-se na tentativa de controlar a micção sem contrair os músculos abdominais, os músculos glúteos e os músculos da parte interna da coxa. Entretanto, não está claro na literatura o envolvimento dos músculos abdominais com o fortalecimento dos músculos pélvicos, pois há hipóteses de que a baixa eficácia do TMAP, em alguns casos, esteja associada à subutilização dos músculos abdominais, o que pode limitar a resposta do músculo pubococcígeo. Esse fato justifica o efeito de outros tratamentos conservadores, como ioga e pilates ^(^ 2020 ^)^ , mas os protocolos encontrados nesta revisão não recomendam o uso desses músculos. 

Devido à especificidade e aos detalhes das medidas descritas nos protocolos do TMAP, fica evidente a relevância do acompanhamento profissional presencial e do esclarecimento com informações escritas de fácil compreensão, seja na forma impressa ou digital. Além disso, para o sucesso efetivo do plano terapêutico, ele deve sempre ser combinado com outras intervenções comportamentais essenciais para o controle gradual da continência, como hábitos alimentares, atividade física e uso do banheiro.

Embora 80% dos protocolos do TMAP incluídos na amostra tenham sido obtidos de estudos controlados e randomizados, a heterogeneidade metodológica dos estudos limitou a possibilidade de comparação dos resultados.

No entanto, os achados desta revisão apresentam importantes implicações clínicas e de pesquisa que apoiam o senso crítico dos profissionais de saúde na tomada de decisões.

Os aspectos destacados nos protocolos, como a intensidade das contrações, o número de sessões, o tempo de relaxamento e as contrações sustentadas, podem ser extremamente úteis no manejo de casos específicos na prática clínica. Além disso, a síntese das informações dos protocolos incluídos neste estudo pode contribuir significativamente para o ensino clínico dessas práticas em cursos de graduação e pós-graduação na área da saúde. Isso favorece o senso crítico dos profissionais, permitindo decisões mais assertivas e incentivando a prática baseada em evidências. A integração desses achados na formação dos enfermeiros fortalece a aplicação das melhores evidências científicas disponíveis na tomada de decisões clínicas, garantindo intervenções mais eficazes e seguras.

## Conclusão

Esta revisão destacou que o TMAP é uma intervenção convencional eficaz, econômica e não invasiva de primeira escolha para o tratamento da incontinência urinária pós-prostatectomia, que deve ser iniciada imediatamente após a remoção do cateter urinário de demora.

Também forneceu visão geral dos aspectos incluídos nos protocolos do TMAP em homens com incontinência urinária pós-prostatectomia, mas apontou a escassez de estudos que descrevem os protocolos em detalhes. Assim, o consenso sobre quais instruções devem ser seguidas com relação ao início do TMAP, posicionamento, tempo de contração/relaxamento, intensidade da contração e duração dos protocolos é questionável.

Em vista dessa falta de detalhes, recomenda-se a observação clínica cuidadosa e o relato adequado do método e dos resultados de qualquer proposta futura na busca da melhor evidência.

Para avançar nas discussões e práticas relacionadas à incontinência urinária pós-prostatectomia, é necessário desenvolver diretrizes de consenso que definam claramente os parâmetros dos protocolos de treinamento. Os resultados obtidos com o mapeamento realizado podem subsidiar a criação de uma lista que reúna as práticas mais citadas na literatura, cuja eficácia possa ser avaliada em futuros ensaios clínicos, com o objetivo de padronizar protocolo de treinamento para os músculos do assoalho pélvico.
